# Experimental phasing opportunities for macromolecular crystallography at very long wavelengths

**DOI:** 10.1038/s42004-023-01014-0

**Published:** 2023-10-12

**Authors:** Kamel El Omari, Ramona Duman, Vitaliy Mykhaylyk, Christian M. Orr, Merlyn Latimer-Smith, Graeme Winter, Vinay Grama, Feng Qu, Kiran Bountra, Hok Sau Kwong, Maria Romano, Rosana I. Reis, Lutz Vogeley, Luca Vecchia, C. David Owen, Sina Wittmann, Max Renner, Miki Senda, Naohiro Matsugaki, Yoshiaki Kawano, Thomas A. Bowden, Isabel Moraes, Jonathan M. Grimes, Erika J. Mancini, Martin A. Walsh, Cristiane R. Guzzo, Raymond J. Owens, E. Yvonne Jones, David G. Brown, Dave I. Stuart, Konstantinos Beis, Armin Wagner

**Affiliations:** 1 Diamond Light Source, Harwell Science and Innovation Campus, -, OX110DE UK; 2 Research Complex at Harwell, Rutherford Appleton Laboratory, Didcot, OX11 0FA UK; 3 Department of Life Sciences, Imperial College London, London, SW7 2AZ UK; 4 National Physical Laboratory, Hampton Road, Teddington, TW11 0LW UK; 5 Charles River Discovery Research Services UK, Chesterford Research Park, Saffron Walden, CB10 1XL UK; 6 Division of Structural Biology, Wellcome Centre for Human Genetics, University of Oxford, Oxford, OX3 7BN UK; 7 Department of Biochemistry, University of Oxford, Oxford, UK; 8 Structural Biology Research Center, Institute of Materials Structure Science, High Energy Accelerator Research Organization (KEK), Tsukuba, Ibaraki 305-0801 Japan; 9 Department of Materials Structure Science, School of High Energy Accelerator Science, The Graduate University of Advanced Studies (Soken-dai), 1-1 Oho, Tsukuba, Ibaraki 305-0801 Japan; 10 Advanced Photon Technology Division, RIKEN SPring-8 Center, Hyogo, 679-5148 Japan; 11 School of Life Sciences, University of Sussex, Falmer, Brighton, BN1 9QG UK; 12 Department of Microbiology, Institute of Biomedical Sciences, University of São Paulo, São Paulo, 05508-000 Brazil; 13 The Rosalind Franklin Institute, Harwell Campus, Oxford, OX11 0FA UK; 14 Present Address: Department of Biochemistry, University of Oxford, Oxford, UK; 15 Present Address: Institute of Biostructures and Bioimaging, IBB, CNR, 80131 Naples, Italy; 16 Present Address: Department of Pharmacy, University of Naples “Federico II”, 80131 Naples, Italy; 17 Present Address: Max Planck Institute of Molecular Cell Biology and Genetics (MPI-CBG), Dresden, Germany; 18 Present Address: Friedrich Miescher Institute for Biomedical Research, Basel, Switzerland; 19 Present Address: Institute of Molecular Biology (IMB), Ackermannweg 4, 55128 Mainz, Germany; 20 Present Address: Department of Chemistry, Umeå University, 901 87 Umeå, Sweden

**Keywords:** X-ray crystallography, X-ray crystallography, X-ray diffraction, X-ray crystallography

## Abstract

Despite recent advances in cryo-electron microscopy and artificial intelligence-based model predictions, a significant fraction of structure determinations by macromolecular crystallography still requires experimental phasing, usually by means of single-wavelength anomalous diffraction (SAD) techniques. Most synchrotron beamlines provide highly brilliant beams of X-rays of between 0.7 and 2 Å wavelength. Use of longer wavelengths to access the absorption edges of biologically important lighter atoms such as calcium, potassium, chlorine, sulfur and phosphorus for native-SAD phasing is attractive but technically highly challenging. The long-wavelength beamline I23 at Diamond Light Source overcomes these limitations and extends the accessible wavelength range to *λ* = 5.9 Å. Here we report 22 macromolecular structures solved in this extended wavelength range, using anomalous scattering from a range of elements which demonstrate the routine feasibility of lighter atom phasing. We suggest that, in light of its advantages, long-wavelength crystallography is a compelling option for experimental phasing.

## Abstract (web summary)

Structural biology has undergone a revolution thanks to cryo-EM and artificial intelligence-based model predictions; nonetheless, experimental phasing continues to be essential. Here, the authors utilize the long-wavelength I23 beamline at Diamond Light Source to solve macromolecular structures using single-wavelength anomalous diffraction techniques, showcasing their proficiency in phasing with lighter atoms.

## Introduction

Structural biology is fundamental to understanding macromolecular functions, their role in diseases, and progress in the development of therapeutic molecules. To date, X-ray crystallography has contributed to the vast majority of protein structures deposited in the protein data bank (PDB)^1^, but structural biology is a fast-expanding field that has recently undergone two major breakthroughs. Before the start of the cryo-electron microscopy (cryo-EM) resolution revolution in 2013^2^, structures of large proteins and assemblies were notoriously more difficult to determine. Cryo-EM has become a key structural technique and can now routinely elucidate atomic details of very large macromolecular machines such as the nuclear pore complex^3^. More recently, the utilization of machine learning techniques by AlphaFold2^4^ or RoseTTAFold^5^ has revolutionized protein model prediction, already having a huge impact on biological research at various levels, for example by providing search models for molecular replacement (MR) to overcome the phase problem in macromolecular crystallography.

Nonetheless, X-ray crystallography remains the major structural technique for protein structure determinations (9839 and 4111 deposited structures in the PDB in 2022 for crystallography and cryo-EM, respectively). Two important factors contribute to this: firstly, structure determination by cryo-EM of macromolecules smaller than 100 kDa can be challenging with preferential orientations in the grid and requires significant computational resources. Secondly, AlphaFold2/RoseTTAFold prediction models need to be validated experimentally. Although protein structure predictions can be very accurate, they remain hypotheses, that can differ from the experimental structures on a global and/or local scale^6^. Accuracy of side chains is key to understanding protein function and for drug discovery. In some cases, the predictions are not accurate enough for molecular replacement techniques, indeed MR search models should not deviate from the actual model more than 1–2 Å (rmsd of Cα atoms) over 50% of the structure^7^. As an example, iterative AlphaFold predictions provided successful molecular replacement models for 87% of the structures solved by single-wavelength anomalous diffraction (SAD) phasing and released in the PDB from 08/12/2021 to 29/06/2022^6^, so more than 10% of these structures still required experimental phasing. In addition, structures that could not be solved and thus are not deposited, could not be considered in this study, hence this percentage is likely to be higher. Of the current ~200 million predictions from AlphaFold2, ~35% are matching experimentally determined structures, and another ~45% are considered to be accurate enough for many applications^8^, resulting in ~20% of structures still requiring phasing. In conclusion, there are still numerous cases where cryo-EM and molecular replacement with available or predicted models will not be successful, thus experimental phasing will be necessary to elucidate the refractory protein structures.

The method of choice for experimental phasing is the SAD technique^9^, which requires collecting one or multiple datasets at a single wavelength close to the absorption edge of a chosen anomalous scatterer. Most SAD experiments exploit the anomalous signal of electron-rich atoms, incorporated either by soaking or more routinely by labeling proteins with seleno-methionine that in some cases may not express nor crystallize as the native protein. However, soaking can affect the isomorphism and diffraction quality of crystals while seleno-methionine labeling can decrease protein yields or be difficult for proteins expressed in eukaryotic systems. Therefore, SAD experiments that do not necessitate any derivatization, such as native-SAD, are very attractive methods for phasing^10,11^.

**Fig. 1:**
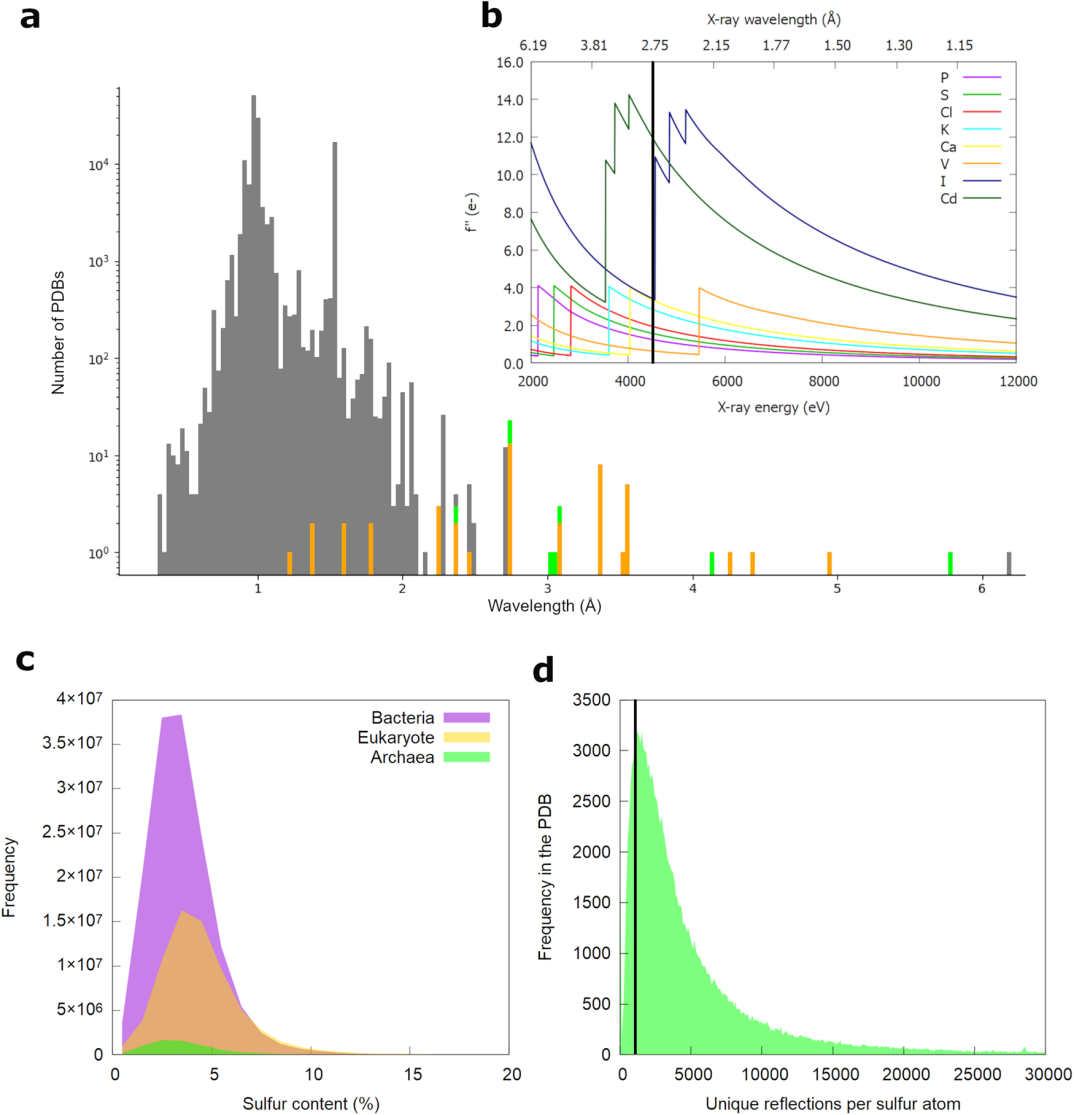
Distribution of wavelengths and sulfur content for native phasing. **a** Distribution of X-ray wavelengths used for crystallographic data collections from the PDB (gray: 132921 PDBs). Datasets from beamline I23 are indicated in orange (45 PDBs), and the ones presented in this paper are colored in green. **b** Variation of coefficient *f*” with X-ray wavelength (or energy) showing typical absorption K-edges (P, S, Cl, Ca, V) and L-edges (I and Cd) accessible at the I23 beamline. The vertical bar indicates the wavelength of *λ* = 2.75 Å (*E* = 4.5 keV). **c** Distribution of sulfur content (%) in the three kingdoms of life, Bacteria (147178681 sequences), Eukaryote (68547281 sequences), and Archaea (6753728 sequences). **d** Distribution of the ratio of unique reflections per sulfur atom in structures deposited in the PDB, the vertical bar indicates a ratio of 1000.

Native-SAD uses light atoms naturally occurring in proteins or bound to proteins. A typical example is the sulfur-SAD experiment (S-SAD) utilizing sulfur atoms present in cysteine and methionine amino acids. On standard synchrotron beamlines, the useful wavelength range is typically between *λ* = 0.7 and 2 Å, with S-SAD experiments being performed at wavelengths of *λ* = 1.77 or 2.06 Å (*E* = 7 or 6 keV, respectively) (Fig. 1a). The anomalous contribution to the scattering factor *f*” increases from shorter wavelengths towards the absorption edge (Fig. 1b). For K-absorption edges, *f*” is approximately 4e^−^ in the absence of white line features which can significantly boost the anomalous signal very close to the edge. L- and M-edges provide significantly larger signals. The sulfur K-edge is found at a wavelength of *λ* = 5.02 Å (*E* = 2.472 keV), so access to longer wavelengths can be greatly beneficial for S-SAD experiments. However, several challenges must be overcome to collect high-quality diffraction data at wavelengths beyond *λ* = 2 Å. Firstly, X-rays are scattered and absorbed by air at longer wavelengths, reducing the attainable signal-to-noise ratio for a given X-ray dose; secondly, larger detectors are needed as the diffraction angles increase with the wavelength, as stated by Bragg’s law (*nλ* = 2dsinΘ). The consequence of using shorter wavelengths for S-SAD is that a reduced anomalous signal is measured (*f*” = 0.7–1e^−^ in the wavelength range of *λ* = 1.77 Å to 2.06 Å compared to 4e^−^ at the S K-edge). To observe the resulting small anomalous differences very accurate data needs to be collected. This has historically been achieved by high multiplicity datasets, often with multiplicity above 100. To avoid the radiation damage that can result from multiple data collections, and depending on the crystal quality and system, successful S-SAD is often only possible with high symmetry crystals and/or merging datasets from multiple isomorphous crystals. A low-dose and multi-crystal approach was shown to enhance the anomalous signal-to-noise ratio while minimizing the signal decay due to radiation damage^12^. In addition to the improvement to data collection strategies, helium environments have also helped to increase native-SAD datasets quality by reducing both the scattering background noise and air absorption of the diffracted X-rays^13,14^.

A more radical approach to reduce air absorption has been used by Beamline I23 at Diamond Light Source. It has been specifically designed to tackle these technical challenges and makes routine native-SAD experiments possible at significantly longer wavelengths^15^. Experiments are run in a vacuum (<10^−7^ mbar) and data is recorded on a large semi-cylindrical Pilatus 12 M detector (Dectris). As a consequence of the vacuum environment, the samples are cooled by conduction rather than a nitrogen cryostream, and dedicated sample holders are used for improved thermal conductivity. An added benefit of collecting crystallographic data in a vacuum is the substantial decrease in background noise in the absence of air scattering, leading to higher signal-to-noise ratios by reducing noise rather than increasing the X-ray flux.

In addition to sulfur, other light element absorption edges are in the wavelength range attainable by the beamline (Ca, K, Cl, and P) (Fig. 1b). Not only can these elements be used for SAD phasing, but anomalous difference Fourier maps can also be used for their detection and localization if external phases are available. These elements of biological importance are found and used in cells in diverse biochemical processes, and their identification and localization in proteins give useful functional insights.

In this study, we report the determination of diverse protein structures by experimental phasing techniques carried out at long wavelengths on beamline I23 at Diamond Light Source using S, Ca, K, Cl, V, I, Cd, and P as anomalous scatterers. The results clearly demonstrate the benefit of native-SAD phasing at long wavelengths, and we suggest that long-wavelength native-SAD should be considered for the experimental phasing of novel targets.

## Results and discussion

### Protein sulfur content for S-SAD

To consider S-SAD as a generic method for phasing, we have analyzed the proteome of the different domains of life (Fig. 1c). The average sulfur content, defined as the percentage of the sulfur-containing amino acids cysteine and methionine in archaea and bacteria is about 3.5% (median 3.2%), whereas in eukaryotes it is slightly higher, with a mean sulfur content of about 4.4% (median 4.1%). Obviously, there are variations in the sulfur content; extracellular proteins tend to be rich in cysteines and disulfides since they are situated in an oxidizing environment. Often domains rather than full-length proteins are studied by X-ray crystallography, making them easy targets. The Bijvoet ratio^16^ has been used as a measure for the expected anomalous signal and a value of 0.6% has originally been proposed as a lower limit to predict the success of SAD phasing experiments^17^. Based on this, a sulfur content of 2% would be enough to measure a useful anomalous signal for S-SAD at *λ* = 2.06 Å, and even a sulfur content of only 0.25% would be sufficient at wavelengths close to the sulfur K-edge (*λ* = 5.02 Å). However, the sulfur content on its own is not a reliable indicator for a successful S-SAD experiment. Terwilliger et al. studied the important parameters contributing to SAD analyses and found that a low number of unique acentric reflections and a high number of anomalous scatterers are detrimental to the success of SAD phasing^18^. Based on 52 S-SAD projects on beamline I23, we simplified the resulting formula to the ratio between the number of unique reflections and that of anomalous scatterers. For successful S-SAD phasing of data collected at a wavelength of *λ* = 2.75 Å on beamline I23, this ratio typically needs to be over 1000 which corresponds to 89% of deposited structures in the PDB (Fig. 1d). Through an analysis of 52 S-SAD projects collected on I23, we successfully solved 41 structures. Among these 41 structures, only 4 had a ratio below 1000, accounting for over 10% of the successful cases. Conversely, out of the 11 failed projects, 8 had a ratio below 1000, representing approximately 72% of the failures. For a given sized protein, a larger number of sulfur atoms will require a correspondingly higher number of unique acentric reflections, hence higher resolution. We have developed a web app that can predict the resolution required for successful S-SAD on the I23 beamline at *λ* = 2.75 Å based on the space group, unit cell parameters, and sulfur content (Yosoku-I23 Resolution Requirement for Phasing, https://diamondi23.anvil.app/).

### S-SAD measurements at long wavelengths

As stated previously, for S-SAD the anomalous signal increases towards the sulfur K-edge. However, due to sample absorption effects, collecting datasets at very long wavelengths is detrimental to the data quality, compromising the anomalous signal. We have used test crystals (insulin, lysozyme, proteinase K, and thaumatin) to determine the optimum wavelength for S-SAD experiments on the I23 beamline (Supplementary Fig. 1 and Supplementary Tables 1–4). A wavelength of *λ* = 2.75 Å (*f*” = 1.6e^−^) was found to be a good compromise and if the crystals have uniform dimensions, it is possible to select a longer wavelength. At longer wavelengths, X-ray absorption from the crystals, sample holder, and surrounding materials is difficult to account for with current data reduction software packages, especially when the shape of the crystal is not uniform. Once the wavelength is set, the typical I23 data collection strategy for native-SAD phasing requires 3 × 360° of data from a single crystal taken at multiple orientations with a multi-axis goniometer with a flux of ~2 × 10^10^ photons s^−1^ from a non-focused beam matching the crystal size. The number of datasets required for structure solution and electron density map interpretation is determined in real-time by evaluating the success of substructure solution and secondary structure identification in electron density maps after each collected dataset. This data collection strategy allows the assessment of radiation damage after each low-dose dataset. Successful experimental phasing can be considered as proof that radiation damage has been minimized during data collection.

We also used test crystals containing sulfur (thaumatin collected at *λ* = 2.75 Å) and zinc (LMO4 collected at *λ* = 1.28 Å) as anomalous scatterers to assess the benefit of our experimental setup. A number of factors might contribute to enhancing the anomalous signal, such as beam stability, the absence of a cryo-stream responsible for sample vibration, or the decreased parallax effect due to the cylindrical shape of the detector but we focused on the reduced background noise of the detector, which is a result of the in-vacuum environment. The sulfur and zinc anomalous peak heights were calculated for different noise levels added artificially. The results demonstrate that the presence of added background noise diminishes the heights of anomalous peaks for both crystals (Supplementary Fig. 2). This effect is more prominent in the LMO4 datasets since it is more weakly diffracting than the thaumatin ones (LMO4 *I*/*σI* = 16.6 vs. Thaumatin *I*/*σI* = 42.2) (Supplementary Tables 5–6). These results show that reduced background noise is clearly benefiting the anomalous signal, and this is even more pronounced for weakly diffracting crystals.

### S-SAD as a routine method

**Fig. 2:**
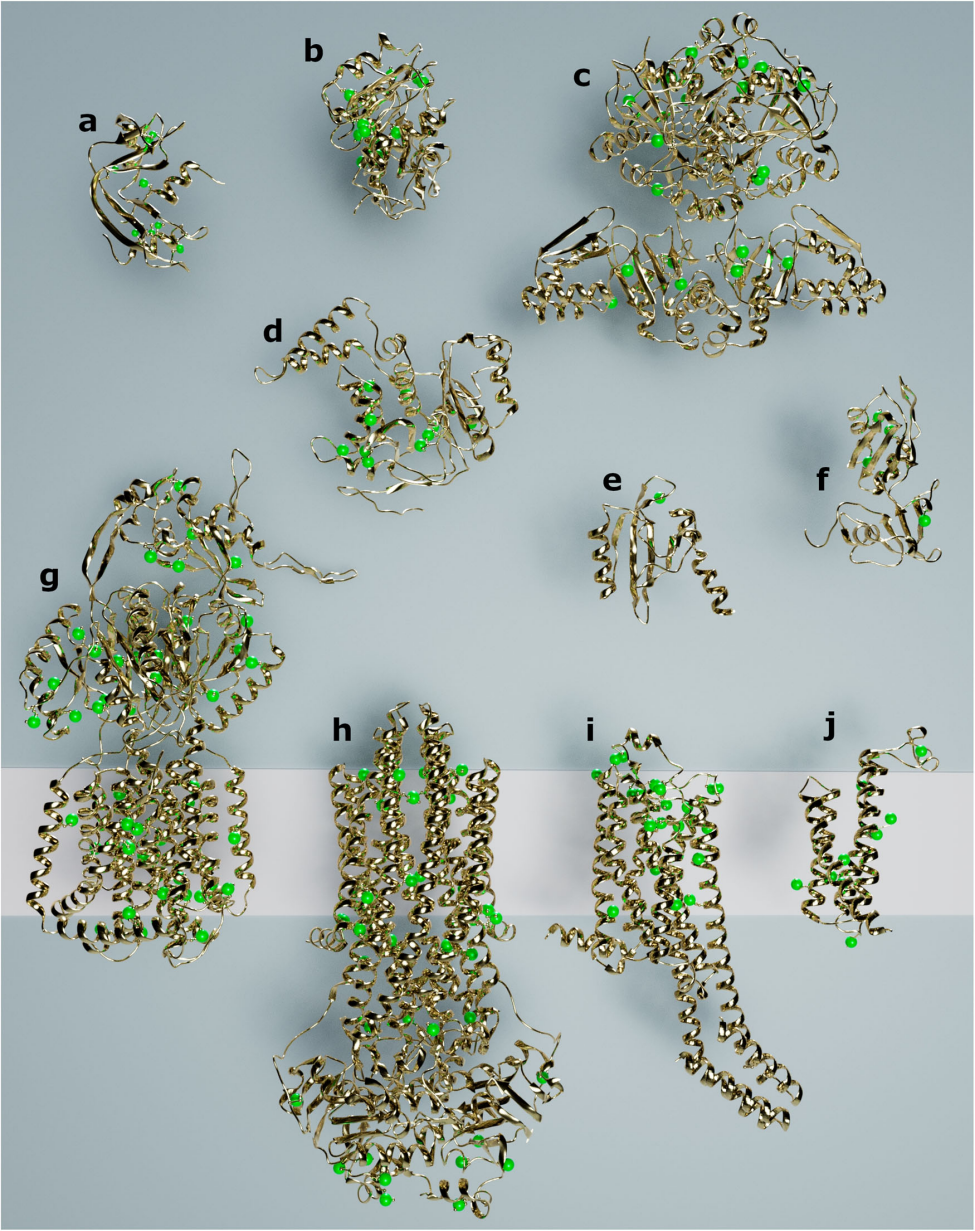
Selected structures solved by S-SAD on the I23 beamline. Protein structures are shown in cartoon representation, sulfur atoms are depicted as green spheres and the pink rectangle in the background represents the cell membrane. **a** RNaseA. **b** PETASE. **c** ThcOx. **d** SseK3. **e** PAS domain. **f** Seb1-RRM. **g** AcrB. **h** McjD. **i** A_2A_R. **j** mPGES.

**Fig. 3:**
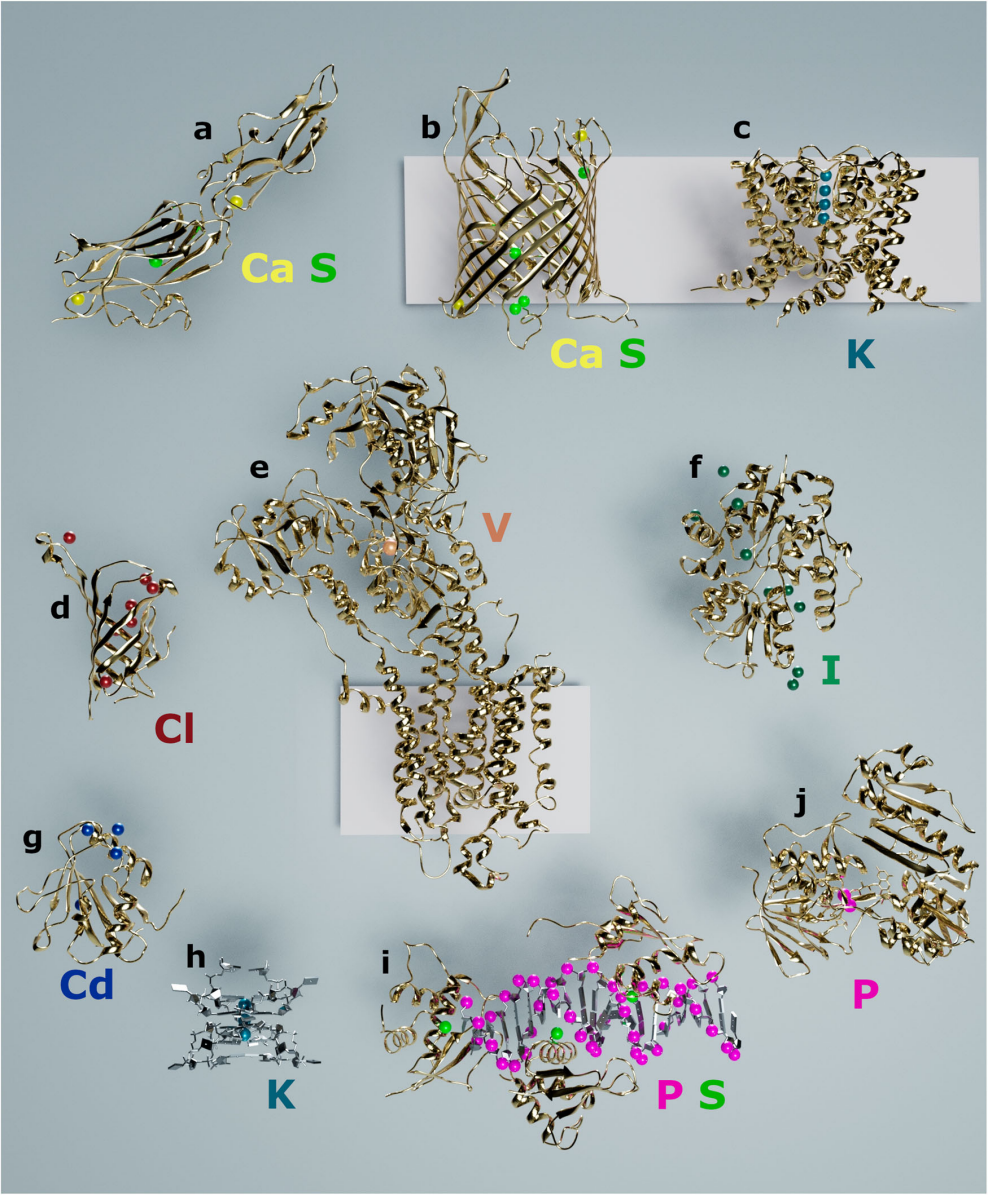
Selected structures solved by native-SAD on the I23 beamline. Protein (gold) and nucleic acids (gray) structures are shown in cartoon representation and anomalous scatterers are represented as spheres of different colors, yellow: calcium, green: sulfur, teal: potassium, red: chlorine, orange: vanadium, dark green: iodine, blue: cadmium, pink: phosphorus. The pink rectangles in the background represent the cell membrane. **a** Fap1. **b** AlgE. **c** NaK2K. **d** Streptactin. **e** SERCA. **f** TauA. **g** Loei River virus GP1. **h** RNA G-quadruplex. **i** IRF4-DNA. **j** BphA4.

**Fig. 4:**
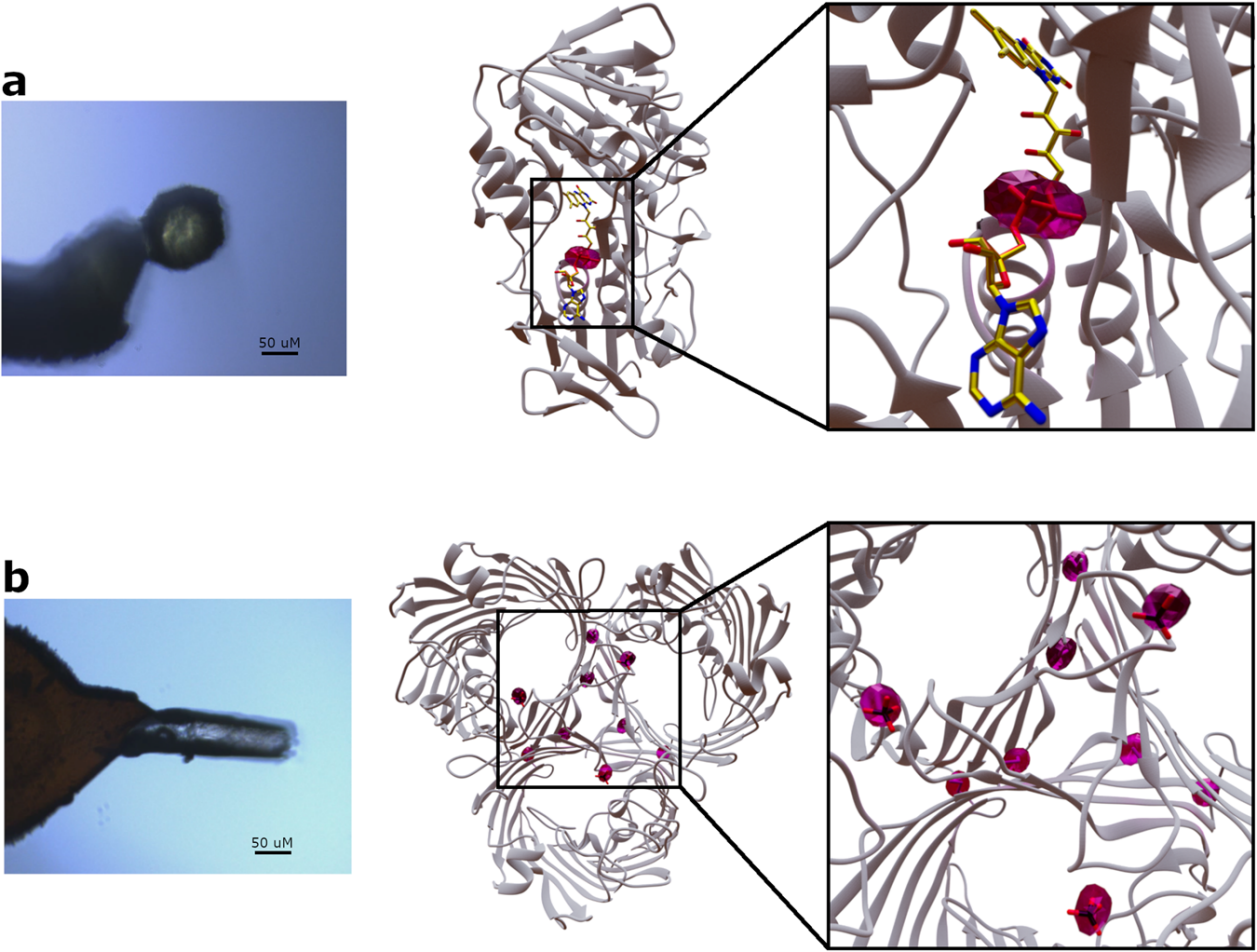
Native SAD with laser-shaped crystals. **a** On the left, spherically shaped crystal of BphA4. On the right, the crystal structure of BphA4 is shown as a cartoon representation and colored in gray while the FAD molecule is depicted as sticks. **b** On the left, a cylindrically shaped crystal of OmpK36. On the right, the crystal structure of the trimeric OmpK36 is shown as a cartoon representation, with sulfate ions and methionine residues as sticks. Phased anomalous difference Fourier maps for BphA4 and OmpK36 are drawn in magenta and contoured at 5 σ.

Using this protocol, we selected 10 projects solved by S-SAD phasing at the beamline, including soluble and membrane proteins (Fig. 2 and Supplementary Table 7). Structures varied in size with molecular weights ranging from 14 to 114 kDa, sulfur contents varied between 0.9 and 6.5%, and diffraction resolution from 1.8 (detector limited) to 3.4 Å. The use of native SAD has also been extended to 9 additional projects where potassium, chlorine, phosphorus, calcium, vanadium, cadmium, and iodine were exploited as main anomalous scatterers. These elements were present as co-factors or in the crystallization conditions (Fig. 3 and Supplementary Table 8). We also report the structure solution of two laser-shaped protein crystals that allow anomalous data collected at wavelengths longer than 4 Å to be improved (Fig. 4 and Supplementary Table 9). Finally, to assess and compare the anomalous signal from these projects, anomalous peak heights from phased anomalous difference Fourier maps are reported in Supplementary Tables 10 and 11.

Soluble proteins such as RNaseA, Petase^19^, ThcOx^20^, or Ssek3^21^ (Fig. 2a–d) represent typical examples that were solved by S-SAD following our protocol, despite ThcOx diffracting to a resolution of 3.1 Å and Ssek3 showing signs of pseudo-translation. The overall multiplicity for these datasets was below 26, which is four times lower than the multiplicity typically needed for S-SAD at shorter wavelengths (100)^10^ (Supplementary Table 7).

As mentioned before, most proteins have a sulfur content sufficient for S-SAD phasing. Amongst the structures solved at the beamline, the lowest sulfur content was found in the PAS domain of the protein codified by the locus tag LIC_11128 from *Leptospira interrogans serovar Copenhageni Fiocruz* L1-130 (Fig. 2e). The domain contains only one sulfur in 116 amino acids (0.9% sulfur content), nevertheless the substructure solution was straightforward in SHELXD^22^ with 360° of data, a resolution of 2.5 Å, completeness of 83% and an overall multiplicity of 11. For refinement purposes, additional datasets were included, resulting in a final multiplicity of 49.2. However, these additional datasets were not necessary for the structure solution. This example shows that low sulfur content is not necessarily a limiting factor for phasing if the anomalous signal can be enhanced at longer wavelengths, here *λ* = 3.09 Å.

To further showcase the capability of in-vacuum long-wavelength beamlines, we determined the structure of the RNA recognition motif (RRM) of Seb1 (Fig. 2f) that was originally solved by S-SAD after collecting datasets from 16 crystals at a wavelength of 1.77 Å on beamline I03 at the Diamond Light Source^23^. A merged dataset with an overall multiplicity of 167 was obtained and led to the sulfur substructure being solved in SHELXD^22^, nevertheless, phase extension to a native resolution of 1.0 Å was needed to get interpretable experimental electron density maps. To confirm the benefit of collecting at long wavelengths, we collected from Seb1-RRM crystals on beamline I23. A single dataset of 360° from a crystal diffracting to 1.8 Å was sufficient not only to solve the substructure but also to solve the structure without any phase extension, despite the low symmetry space group (*C*2), low overall completeness (65%) and the 27-fold lower multiplicity of only 6. This case clearly shows that data collection at longer wavelengths requires substantially reduced overall multiplicity. Solving a structure from a single crystal with low multiplicity is advantageous for multiple reasons: the number of crystals might be limited; crystals may not be isomorphous and cannot be merged or they may be especially susceptible to radiation damage.

Since integral membrane proteins are notoriously difficult to study, we show here that S-SAD (with sulfur as the only anomalous scatterer) was successful in phasing the α-helical membrane proteins AcrB^24^, McjD^25^, A_2A_R, and mPGES (Fig. 2g–j). mPGES, diffracting to 1.77 Å resolution, was straightforward to solve even with a low overall multiplicity of 17.5. In contrast, AcrB, McjD, and A_2A_R diffracted to only modest resolution: 3.4, 2.8, and 2.9 Å respectively (with corresponding molecular weights of 115, 65, and 50 kDa). A_2A_R was previously solved with long-wavelength native phasing at X-ray free-electron lasers at 2.65 Å resolution (detector limited)^26^, using a serial crystallographic approach with samples delivered by a high viscosity injector. In total, 199136 images from crystals with an average size of 35 × 35 × 5 µm were collected at the SwissFEL at a wavelength of *λ* = 2.71 Å, and automatic phasing was successful using data from 50000 crystals. On beamline I23, a single crystal of A_2A_R 20 × 20 × 5 µm (18,000 images) was sufficient for successful S-SAD phasing. Long-wavelength native phasing at X-ray free-electron lasers may be useful if crystals cannot be optimized to larger sizes, are very susceptible to radiation damage, or need to be studied at room temperature, otherwise it is easier and more practical to collect at long wavelength at beamline I23. AcrB was possibly one of the most challenging projects due to its large sulfur substructure (45 sulfur atoms) and low resolution (3.4 Å) resulting in a ratio of unique reflections over a number of anomalous scatterers of 650. It required the collection of five datasets of 360° each, reaching an overall multiplicity of 86.9, all from a single crystal because of non-isomorphism. For McjD, two crystals were needed totaling six datasets of 360° and an overall multiplicity of 54. These examples show that S-SAD is not necessarily limited to well-diffracting crystals and the method is applicable even to more difficult membrane proteins.

### Other-SAD at long wavelengths

About a third of proteins are bound to a metal cofactor, with zinc and iron regularly used for phasing. Calcium is used less often because its K-edge is located at a longer wavelength (*λ* = 3.07 Å), typically not accessible at standard MX beamlines. A domain of the accessory Sec-dependent serine-rich glycoprotein adhesin from *Streptococcus oralis* (Fap1) was solved by Ca-SAD on beamline I23 from two calcium ions and 1080° of data to a resolution of 2 Å (Fig. 3a). Compared to sulfur, the calcium anomalous signal is stronger and more efficient for phasing (Supplementary Table 11). Calcium plays an important role in many cellular processes, so it can be easily used instead of, or combined with sulfur for phasing, as in the case of the β-barrel membrane protein AlgE (Fig. 3b). AlgE crystallized in a low symmetry space group (*C*2) and contained 6 sulfur and 2 calcium atoms. For successful phasing, it was necessary to merge datasets from 2 crystals (a total of 7 × 360°). The substructure could be found readily but increasing the multiplicity to 26.7 was necessary to obtain interpretable electron density maps. From our experience, proteins with secondary structures mainly composed of β-strands tend to need more data to obtain interpretable initial maps.

Where sulfur is not present in the protein, other absorption edges can be reached on the beamline I23. We have solved the K^+^ selective transporter NaK2K using K-SAD (potassium K-edge = 3.43 Å) with four K^+^ ions located within the selectivity filter of the membrane protein with strong anomalous peak heights >30 σ^27^ (Supplementary Table 11) (Fig. 3c). A second example of a protein without any sulfur atoms is the streptavidin mutant Streptactin XT^28^ (Fig. 3d). Data were collected at *λ* = 2.75 Å from crystals that diffracted to better than 1.8 Å resolution. The structure could easily be solved with seven chloride ions bound to the protein although the data was not collected at the chlorine absorption edge (Cl K-edge = 4.39 Å). To the best of our knowledge, this is the first example of Cl-SAD.

Within the I23 accessible wavelength range, absorption edges of non-physiological elements can also be used. These elements are either incorporated in protein ligands, such as vanadium in vanadate or are present in the crystallization buffer. We have previously shown that V-SAD is a rapid method to obtain experimental phases for protein structure determination^29^. A single vanadium, bound as a reaction mechanism inhibitor (VO_3_^−^), was enough to solve the 110 kDa membrane protein SERCA diffracting to 3.1 Å resolution (Fig. 3e). Iodine has been used to phase protein structures for example with the magic triangle^30^ and is sometimes found in crystallization conditions, but the three iodine L-edges are located between *λ* = 2.38–2.72 Å (Fig. 1b). Hence a beamline that can access longer wavelengths is more suitable to get the optimum iodine anomalous signal (*f*” > 10e^−^). The protein TauA was crystallized in a solution containing 200 mM NaI, 14 iodine ions were bound to two TauA molecules present in the asymmetric unit, allowing the structure to be swiftly solved by I-SAD^31^ (Fig. 3f). Another electron-rich element that was used on the beamline is cadmium, again found in the crystallization condition of the Loei River virus GP1 glycoprotein (Fig. 3g). Data from GP1 crystals were collected at *λ* = 2.75 Å (Cd L-edges = 3.08–3.53 Å) giving diffraction to 3 Å resolution, with four Cd atoms bound to the protein^32^. With a *f*” > 10e^−^, cadmium provides a strong anomalous signal even at low resolution.

Finally, phosphorus is an essential element in biology and is found in nucleic acids and some protein co-factors such as NADPH and FADH. There are very few nucleic structures solved by P-SAD, which can be explained by two main reasons. Firstly, phosphorus atoms in the nucleic acid backbone tend to have higher B-factors than for example main chain protein atoms, as they are exposed on the surface of the molecule, and larger atomic displacements make substructure determination more difficult. Secondly, nucleic acid crystals usually have small unit cells, hence a low number of unique reflections, and a high number of anomalous scatterers (one per nucleotide). From our experience of solving nucleic acid structures at medium resolution, an additional anomalous scatterer is needed, even if a strong phosphorus anomalous signal is measured. The Pseudorabies virus RNA G-quadruplex and the i-motif of the human telomeric sequence were solved with potassium and bromine, respectively^33^ (Fig. 3h). These initial phases were used to locate the phosphorus atoms and improve the experimental phases for model building. Being able to solve the structure with potassium and not phosphorus shows again that the ratio of unique reflections over a number of anomalous scatterers is critical for structure determination. In the case of nucleic acid—protein complexes, asymmetric units are larger because of the presence of proteins yielding a larger number of unique reflections per anomalous scatterer. Hence, the structure of such complexes can be elucidated; as in the case of IRF4, where both phosphorus and sulfur atoms contributed to the phasing power^34^ (Fig. 3i).

### Managing X-ray absorption at long wavelengths

Despite the evident benefit of long wavelengths for experimental phasing, the protocol can be further improved by managing the effect of X-ray absorption from the samples, either by introducing analytical sample absorption corrections or by machining the crystal sample to a uniform shape, such as a sphere or a cylinder. The first method requires an accurate measurement of the sample shape, which can be obtained from X-ray tomography experiments. An absorption factor, based on the path length through the different sample materials (crystal, sample holder, and surrounding materials) and their absorption coefficients, can be applied for each reflection as the basis of the analytical absorption correction. The second method applies laser shaping of the crystal to remove all non-diffracting materials and define more regular path lengths through the crystals^35^. This method has the added advantage of choosing the crystal size, as smaller crystals absorb less X-rays at longer wavelengths. Crystals of BphA4 complexed with FAD were shaped as spheres at SPring-8 (Japan) and collected at the phosphorus edge (*λ* = 5.76 Å) on beamline I23 (Fig. 3j and Supplementary Table 9). At this wavelength, the anomalous signal from sulfur is negligible. Only the anomalous signal from the two phosphorus atoms of the FAD molecule was present to successfully phase BphA4, despite the low resolution of 3.7 Å (detector limited) and a low overall multiplicity of 12. Since the two phosphorus atoms are close to each other, they behave as a super-phosphorus with an anomalous peak height of 39.7 σ (Supplementary Table 11) (Fig. 4a). We also laser-shaped crystals of the β-barrel membrane protein Ompk36^36^ as cylinders. Ompk36 crystallized as a trimer, with 2 sulfur atoms per monomer and 3 additional sulfate ions bound to the trimer. Six datasets of 360° each (multiplicity of 22.3) were collected from a single crystal at a wavelength of 4.13 Å and were sufficient for S-SAD phasing (Fig. 4b and Supplementary Tables 9–10). The resolution of the structure was limited to 2.7 Å due to the detector resolution limitation at this wavelength. As mentioned before, β-barrel membrane proteins are more challenging to solve and our attempts to solve the structure with our standard protocol (i.e., not laser-shaped) were unsuccessful. This example shows how long wavelengths can be crucial in solving a difficult structure. The full potential of the beamline can be achieved if the absorption effects are corrected or equalized and the crystal size is decreased. Laser shaping provides an enormous advantage for long-wavelength phasing and helps with extracting the optimum anomalous signal from sulfur, phosphorus, and other elements. Laser shaping can also be applied to determine the identity and possibly the oxidation state of light metals. At the longest wavelengths available, even a large detector like the Pilatus 12 M is limited to about 3.7 Å resolution (Supplementary Fig. 3), but since the multiplicity required for phasing is very low, the same crystal can also be measured at shorter wavelengths to obtain higher resolution for structure refinement.

In conclusion, the beamline I23 at Diamond Light Source significantly extends the available wavelength range (*λ* = 1.1–5.9 Å) for anomalous experiments on macromolecular crystals. A variety of absorption edges can be utilized for experimental phasing and element identification, including K-edges (Zn, Cu, Ni, Co, Fe, Mn, Cr, V, Ca, K, Cl, S, P), L-edges (I, Cd, Ag, Pb), and M-edges (Pb, Hg, Au, Pt). While certain experiments, such as Zn-SAD, can be performed on standard beamlines, conducting the same experiment on I23 would yield superior results due to its significantly lower background, resulting in data of higher quality. We demonstrate that long-wavelength native-SAD has become a very compelling technique, where a low multiplicity dataset from a single crystal can be sufficient for successful structure determination, making it a general vehicle of choice for experimental phasing. This capability is particularly advantageous for projects with limited crystal availability that require experimental structure solutions. Crystals sensitive to radiation damage also benefit from the I23 beamline instrument due to its low standard flux and non-focused beam, allowing for the collection of multiple sweeps of 360° data. Moreover, the large beam size (up to 500 × 500 μm) enables the entire crystal to be exposed, resulting in increased recorded intensity. Typically, once the multiple datasets have been merged, the recorded resolution on the I23 beamline is comparable to that of standard beamlines. However, it is important to note that at long wavelengths, the resolution can be limited by the detector, as illustrated in Supplementary Fig. 3. Se-SAD (*λ* = 0.97 Å, *f*” = 4e^−^) is a widely used experimental phasing method, and although the I23 beamline cannot reach such short wavelengths, the same *f*” value can be obtained at 3.1 Å wavelength (close to the Se L-edge: *λ* = 7.49 Å). However, this does not account for the Se K-edge white line. Hence, a standard beamline is more suitable, especially for Se-MAD experiments. Nevertheless, one must consider if Se labeling is necessary when S-SAD can be performed with native crystals. In addition, native-SAD data provides accurate locations of anomalous scatterers: sulfur positions can assist with assigning the protein sequence with the help of cysteines and methionines, and positions of other scatterers can help with the identification of co-factors or metal ions to improve the quality of deposited models. In this study, we show that the increased wavelength range not only boosts the anomalous signal for sulfur and phosphorus, but additional elements, like calcium, potassium, vanadium, or chlorine can now be routinely considered for experimental phasing experiments. We are currently establishing protocols to use analytical absorption corrections and laser shaping to deal with the increased sample absorption. This will improve data quality at the longest wavelengths further to exploit the full potential of this method.

## Methods

### Sulfur content

Briefly, sulfur content analysis of taxonomic rank protein databases was performed by downloading the databases from the EBI uniprotkb server and parsing each sequence for the number of cysteine or methionine residues as well as recording sequence length. These values were then used to determine the percentage of sulfur-containing residues and averaged over all proteins in the database. The code written in Python 3, is available at https://github.com/co2e14/SContent.

To obtain the number of reflections per S atom for structures in the PDB, the Python pypdb package was used. Briefly, the PDB was searched for all structures determined by X-ray diffraction. For each of these structures, the deposited sequence containing each chain and molecule was used to identify the number of cysteine and methionine residues, and therefore number of sulfur atoms in the asymmetric unit. Additionally, the number of Miller indices was extracted from each PDB ID. Using these two values, the ratio of reflections per S atom is given by: ratio = number of miller indices/number of S atoms. The code written in Python 3, is available at https://github.com/co2e14/pypdb.

### Beamline design

The beamline layout has been described previously^15^. The overall beamline wavelength range is *λ* = 1.1–5.9 Å. To avoid the attenuation of long-wavelength X-rays by air, the endstation is directly linked to the storage-ring vacuum and operates at pressures <10^−7^ mbar. All components of the endstation, the sample environment including the goniometer and detector operate in a vacuum. The multi-axis goniometer in inverse-kappa geometry was designed and assembled by the UK Astronomy Technology Center (UK, ATC). A conductive cooling link keeps the goniometer head at temperatures below 55 K. The large semi-cylindrical Pilatus 12 M area detector (DECTRIS, Baden, Switzerland) has been specifically designed for in-vacuum long-wavelength crystallography. It consists of 120 Pilatus2 modules arranged in a semi-cylindrical geometry of 24 banks of five modules. The detector covers an angular range of 2Θ = 40.3° and +/−100° along the cylinder axis and the central meridian respectively. The maximum resolution ranges from *d*_min_ = 0.73 Å at a wavelength of *λ* = 1.1 Å to 4.05 Å at *λ* = 5.9 Å (Supplementary Fig. 3). All modules have been calibrated for long wavelengths in ultra-high gain settings with the lowest detector threshold at 1.8 keV.

To keep the sample at cryogenic temperatures, cooling relies on thermal conductance and two pulse-tube coolers with a base temperature of 8 K that keep the goniometer head, sample changer and sample hotel below 55 K. The transfer of crystals from liquid nitrogen to the in-vacuum endstation is possible with a dedicated cryo-transfer system that has been designed at the Diamond Light Source based on similar devices used in cryo-EM^37^. For additional details about the beamline and the process of proposal submission, please visit the following webpage: https://www.diamond.ac.uk/Instruments/Mx/I23.html.

### Protein production and crystallization

Protein production, purification, and crystallization were previously reported for the projects: RNaseA^38^, Petase^19^, ThcOx^20^, Ssek3^21^, ACRB^24^, MCJD^39^, A_2A_R^40^, mPGES^41^, Ompk36^36^, AlgE^42,43^, Seb1-RRM^23^, NaK2K^27^, SERCA^29^, TauA^31^, Loei River virus GP1^32^, RNA G-quadruplex and the i-motif of human telomeric sequence^33^, IRF4^34^, BphA4^44,45^, LMO4^46^.

The crystallized streptavidin mutant^28^ co-eluted as a low-abundance contaminant protein during the purification of another protein of interest presenting a 3c protease cleavable Twin-Strep tag, and likely leaked from the Streptactin column used for affinity. The expression, purification, and crystallization protocol for the protein of interest was as follows. HEK293S GnTI^–^ TetR mammalian cells stably expressing the protein of interest were grown as suspension cells and induced when the cell density was at 3 × 10^6 ^ml^−1^ with 1 μg ml^−1^ doxycycline for 48 h at 37 °C. After induction, the cells were harvested and the supernatant was dialyzed against 50 mM Tris, pH 8, and 200 mM NaCl, using a QuixStand benchtop system (GE Healthcare) connected to a 60 cm Xampler Cartridge (GE Healthcare) with a 5-kDa nominal MWCO. The sample was then loaded overnight with recirculation onto a 5 ml Streptactin XT High Capacity column (IBA Lifesciences). The protein of interest (together with the contaminating streptactin) was then eluted from the StreptactinXT column in a buffer containing 50 mM Tris, pH 8, 200 mM NaCl, 50 mM biotin. Size-exclusion chromatography was in turn performed in 50 mM Tris, pH 8, 200 mM NaCl using a Superdex 200 Increase 10_300 GL column (GE Healthcare): since the molecular weight of the Streptactin is very close to one of the proteins of interest, size-exclusion chromatography did not remove the contamination, neither the contamination was evident on SDS-PAGE for the same reason and for the low abundance of Streptactin. The SEC fractions corresponding to the protein of interest (+streptactin) were concentrated to 4.5 mg ml^−1^, and crystallization screenings were performed using the sitting-drop vapor diffusion method at 22 °C. Crystals grew in 3 M sodium chloride, 0.1 citric acid, pH 3.5.

Residues 2-115, from *Leptospira interrogans serovar Copenhageni Fiocruz* L1-130 (LIC_11128 gene) expressed and purified with a N-terminal His-tag in the pOPINF vector using standard OPPF methods previously described^47^. The purified recombinant protein (28 mg ml^−1^) crystallized in 0.1 M BIS-Tris pH 5,5, 17% *w*/*v* PEG 10,000, and 0.1 M Ammonium acetate from JCSG-plus™ crystallization kit (Molecular Dimensions). Before X-ray diffraction data, 25% glycerol was added to the crystallization condition as a cryoprotectant.

Starting Fap1 construct comprised residues 251–705 of accessory Sec-dependent serine-rich glycoprotein adhesin from *Streptococcus oralis* (NCBI reference sequence ID: WP_000466186) was cloned into pOPINN-GFP (addgene plasmid #53541). The construct was expressed in *E. coli* (strain BL21) and grown in autoinduction media. Cells were lysed 40 mM Hepes pH 7.5, 500 mM NaCl, 45 mM imidazole using a constant flow cell disrupter. The sample was purified using Nickel affinity and size-exclusion chromatography with a 3C protease step to cleave the GFP tag (1:25 3 C:Fap1 molar ratio). Sparse matrix crystal screens were set up at 20 mg ml^−1^ and crystals appeared after three months in 0.1 M BIS-Tris 5.5, 25% *w*/*v* PEG 3350. It was not possible to solve the phase problem using molecular replacement however differential scanning fluorimetry indicated that calcium had a stabilizing effect, and therefore may be useful for phasing the crystals. The extensive time between setting up the crystal plate and crystallogenesis indicated that protein degradation could be present. Crystals were transferred to beamline I23 to attempt to phase using a calcium anomalous signal. Upon solving the crystal structure, it became apparent that a fragment representing residues 372 to 624 was present, with the remaining residues absent.

The test crystals used for the wavelength optimization experiments were ferritin, insulin, lysozyme, proteinase K, and thaumatin. These crystals are known to diffract beyond the resolution edge of the detector (1.8 Å resolution at *λ* = 2.75 Å). All test proteins were purchased from Sigma-Aldrich. Insulin powder (Sigma I5500) was dissolved to 25 mg ml^−1^ in 50 mM Na_2_HPO_4_ pH 10.5 and 10 mM EDTA. Crystals grew in a 20% ethylene glycol solution. Lysozyme (Sigma, 62971) was dissolved in 10 mM sodium acetate pH 3.8 to a concentration of 50 mg ml^−1^ and crystallized in 100 mM sodium acetate, pH 4.6, 1 M sodium chloride, 25% ethylene glycol. Proteinase K (Sigma, P2308) was dissolved in 25 mM HEPES pH 7.5 at a concentration of 40 mg ml^−1^. The crystallization condition consists of 1.2 M ammonium sulfate, 0.1 M Na cacodylate pH 6.2, and 25% glycerol. Finally, thaumatin (Sigma, T7638) was dissolved in water with a final concentration of 50 mg ml^−1^ and crystallized in 50 mM ADA, pH 6.8, 600 mM potassium sodium tartrate (dissolved in saturated DTNB water) and 20% glycerol.

### Test crystals data collection and processing

The range of energies used was 3.09 to 2.47 Å (4.0 keV to 5.0 keV in increments of 0.25 keV). Data collection was interleaved between the 5 different energies, with 360 of data being collected for each wavelength at 30 wedges at a set transmission determined during screening. The beam size was adjusted to the size of the crystal and datasets were collected with an exposure of 0.1 s and an oscillation to 0.1° using a flux ranging from 5.10^9^ to 2.5.10^10^ photons s^−1^.

Datasets were processed with XDS/XSCALE^48^ (Supplementary Tables 1–4). AIMLESS in CCP4^49^ was used to obtain a mtz file for molecular replacement in PHASER^50^ using the protein models ferritin (PDB: 2WO0), insulin (PDB: 3I40), lysozyme (PDB: 2LYS), proteinase K (PDB: 2ID8), thaumatin (PDB: 1RQW). Following this, SHELXC^48^ and ANODE^51^ were used to generate phased Fourier anomalous difference maps and compare the anomalous signal from different wavelengths.

### Detector background noise analysis

A thaumatin dataset (180°) was collected at *λ* = 2.75 Å to measure the sulfur anomalous signal, while the LMO4 dataset (180°) was collected at *λ* = 1.28 Å to measure the zinc anomalous signal (LMO4). The beam size was adjusted to the size of the crystals and both datasets were collected with an exposure of 0.1 s and an oscillation of 0.1°. A Python script (Supplementary Note 1) was used to add Poisson noise background scattering to the existing dataset images at rates of 0.25, 0.5, 1, 2, and 4. The datasets were then processed with XIA2-DIALS^52^ and molecular replacement was carried out in PHASER with the search models PDB: 1RQW and 1RUT for thaumatin and LMO4, respectively. SHELXC^22^ and ANODE^51^ were used to generate phased Fourier anomalous difference maps to compare the anomalous peak heights in the presence of the different detector background noise levels.

### Laser shaping crystals

BphA4 and Ompk36 crystals were laser shaped into spheres and cylinders respectively at SPring-8 (Japan)^35^. The wavelength of the deep-UV laser was 193 nm and the pulse duration to 1 ns (pulse power: 1 uJ, repetition: 1 kHz). Crystals were mounted on a high-precision single-axis goniometer and kept cooled at 100 K with a nitrogen cryostream.

### Data collection, structure solution, and refinement

All datasets were recorded on the beamline I23 at the Diamond Light Source (Didcot, UK) at wavelengths ranging from 2.26 to 5.6 Å. Typically, a single crystal was sufficient to collect three datasets of 360° on the semi-cylindrical Pilatus 12 M (Dectris) with an exposure of 0.1 s and over an angle of 0.1°. The multi-axis goniometer was used to offset Kappa and Phi angles to minimize hardware systematic errors, and increase true multiplicity and completeness. The beam size was set to match the crystal size with a typical flux of 2 × 10^10^ photons s^−1^. Datasets were processed either with Xia2 DIALS^52^, or XDS/XSCALE^48^ and converted with AIMLESS^49^. Substructures and structures were solved with HKL2MAP^22,53^, CRANK2^54,55^, or PHENIX.AUTOSOL^56^. In some projects, the substructure was obtained with HKL2MAP, and the sites were used in CRANK2 or PHENIX.AUTOSOL to obtain initial experimental electron density maps. Automatic model building was done with BUCCANEER^57^ and further manual model building in COOT^58^. Refinement was performed with PHENIX.REFINE^56^ or REFMAC5^59^. All data processing and refinement statistics are shown in Supplementary Tables 7–9. SHELXC^48^ and ANODE^51^ were used to generate phased anomalous difference Fourier maps from each dataset (Supplementary Tables 10–11).

### Reporting summary

Further information on research design is available in the Nature Portfolio Reporting Summary linked to this article.

### Supplementary information

Supplementary Information

Reporting Summary

## Data Availability

Coordinates and structure factors have been deposited in the Protein Data Bank, PDB codes can be found in Supplementary Tables 7–9. All other data are available from the corresponding author on reasonable request.

## Code availability

The codes to analyze the sulfur content and the number of reflections per S atom from structures in the PDB are available at: https://github.com/co2e14/SContent and https://github.com/co2e14/pypdb.
