# Supplementary material for: Experimental phasing opportunities for macromolecular crystallography at very long wavelengths
Source: Commun Chem. 2023 Oct 12;6:219. doi: 10.1038/s42004-023-01014-0 (PMC10570326; doi:10.1038/s42004-023-01014-0)
Supplement: Supplementary file 1 — Supplementary Information [file 42004_2023_1014_MOESM1_ESM.pdf]

Supplementary figures

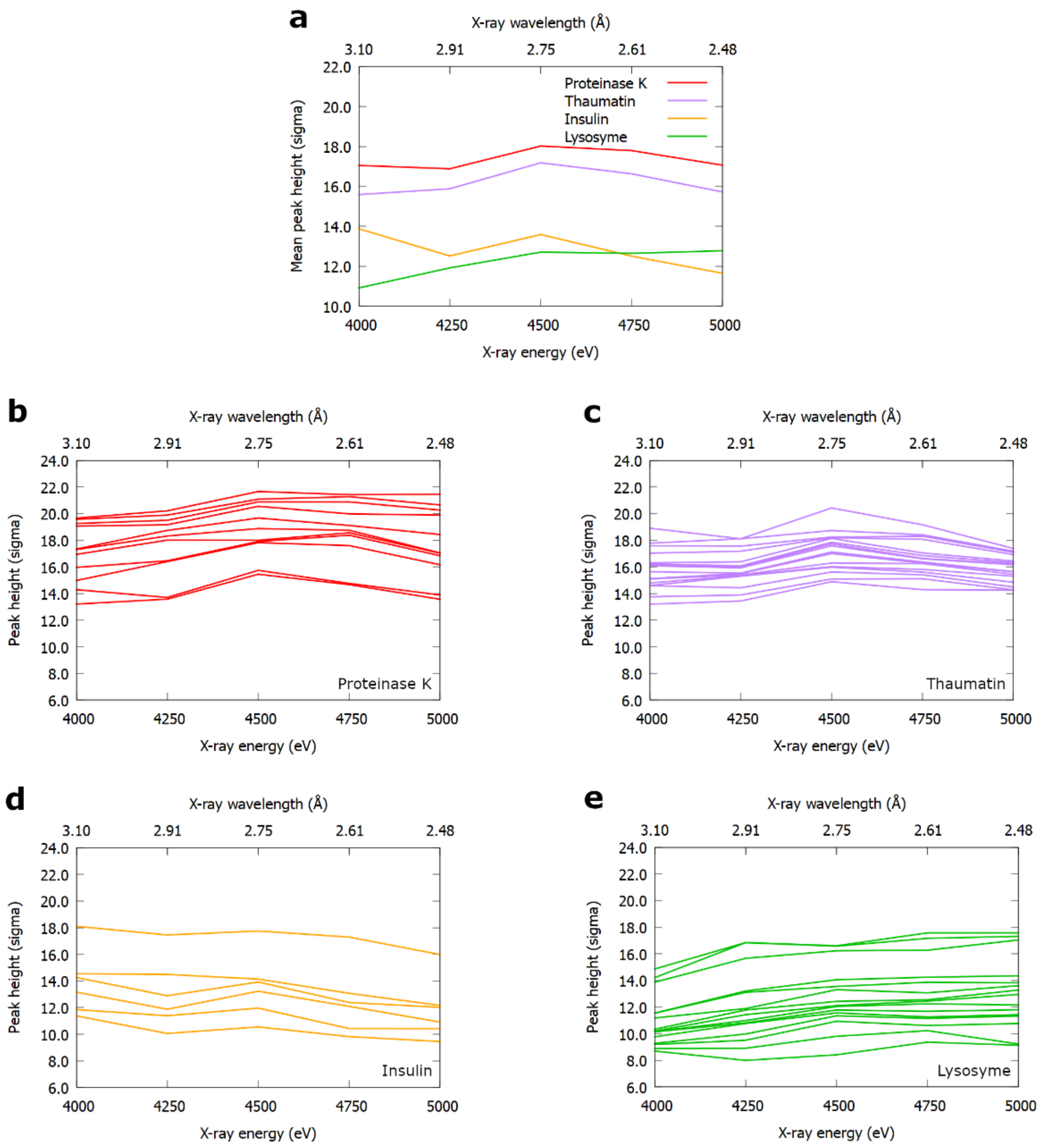

Supplementary Figure 1: Variation of the anomalous mean peak height with X-ray wavelength (or energy) on the I23 beamline. (a) Mean anomalous peak heights for all sulfur atoms and anomalous scatterers were calculated at 3.10, 2.98, 2.75 and 2.48 Å wavelengths for four test crystals. Individual anomalous peak heights are shown as a function of X-ray wavelength (or energy) for each test crystal: proteinase K in red (b), thaumatin in purple (c), insulin in yellow (d) and lysozyme in green (e).

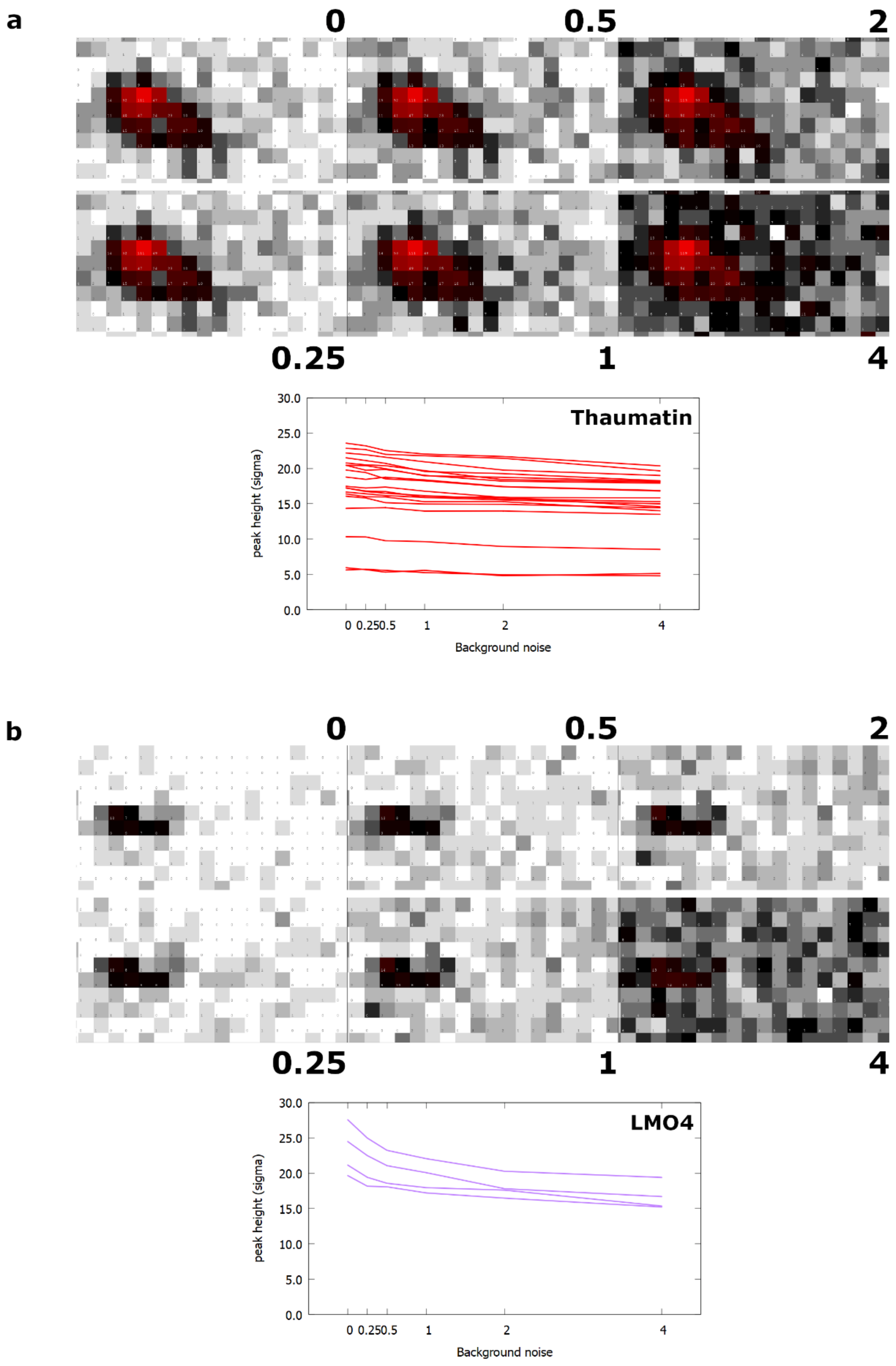

Supplementary Figure 2: Effect of detector background noise on anomalous peak heights. (a) Top- Example of different noise levels (0, 0.25, 0.5, 1, 2, 4) around a diffraction spot from a thaumatin crystal. Bottom- Sulfur peak heights measured from a thaumatin crystal with different detector background noise levels. Each line corresponds to one sulfur atoms. (b) Same as (a) but using a LMO4 crystal and measuring the peak heights of the four intrinsically bound zinc ions.

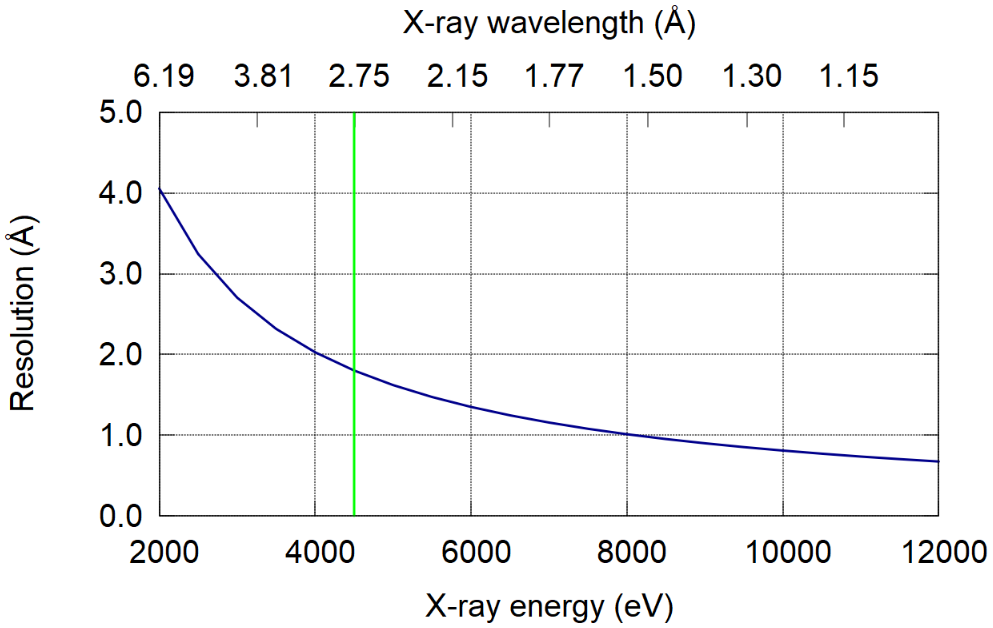

Supplementary Figure 3: Variation of the maximum achievable resolution with X-ray wavelength (or energy) on the I23 Beamline. The detector size of the Pilatus 12M (Dectris) is limiting the resolution at longer wavelengths. At the preferred wavelength of 2.75 Å for S-SAD experiments (vertical green bar), a maximum resolution of 1.8 Å can be recorded.

Supplementary Tables:

	Proteinase K				
Energy (keV)	4	4.25	4.5	4.75	5
Wavelength (Å)	3.099	2.917	2.755	2.610	2.479
Resolution range (Å)	48.17 - 1.99 (2.06 - 1.99)	48.17 - 1.87 (1.94 - 1.87)	48.17 - 1.79 (1.86 - 1.79)	48.17 - 1.79 (1.86 - 1.79)	48.17 - 1.79 (1.86 - 1.79)
Space group	*P* 4_3 2_1 2				
Unit cell dimensions (Å,°)	68.12 68.12 102.08 90 90 90	68.12 68.12 102.08 90 90 90	68.12 68.12 102.08 90 90 90	68.12 68.12 102.09 90 90 90	68.12 68.12 102.09 90 90 90
Total reflections	240938 (7867)	277663 (9383)	338553 (17069)	364370 (26360)	379025 (27520)
Unique reflections	28639 (2374)	33334 (2837)	39407 (3464)	40058 (3678)	40499 (3754)
Multiplicity	8.4 (3.3)	8.3 (3.3)	8.6 (4.9)	9.1 (7.2)	9.4 (7.3)
Completeness (%)	91.43 (75.56)	88.66 (75.07)	92.57 (81.99)	94.03 (87.01)	95.04 (88.71)
Mean I/sigma(I)	18.88 (6.94)	18.47 (5.67)	19.81 (6.79)	20.67 (7.93)	20.55 (7.47)
Wilson B-factor (Å²)	20.09	19.11	17.81	17.49	17.12
R-merge	0.0785 (0.1611)	0.0747 (0.1820)	0.0702 (0.1827)	0.0681 (0.1868)	0.0686 (0.2056)
R-meas	0.0829 (0.1914)	0.0788 (0.2156)	0.0739 (0.2044)	0.0717 (0.2015)	0.0722 (0.2213)
R-pim	0.0260 (0.1015)	0.0245 (0.1134)	0.0227 (0.0886)	0.0218 (0.0736)	0.0218 (0.0799)
CC1/2	0.997 (0.958)	0.998 (0.944)	0.998 (0.969)	0.999 (0.979)	0.999 (0.974)

Supplementary Table 1: Data collection statistics for Proteinase K. Numbers in parentheses refer to the highest resolution data shell.

	Thaumatin				
Energy (keV)	4	4.25	4.5	4.75	5
Wavelength (Å)	3.099	2.917	2.755	2.610	2.479
Resolution range (Å)	54.05 - 1.99 (2.06 - 1.99)	54.05 - 1.87 (1.94 - 1.87)	54.06 - 1.79 (1.86 - 1.79)	54.06 - 1.79 (1.86 - 1.79)	54.06 - 1.79 (1.84 - 1.79)
Space group	*P* 4_1 2_1 2				
Unit cell dimensions (Å,°)	57.92 57.92 150.40 90 90 90	57.92 57.92 150.41 90 90 90	57.93 57.93 150.43 90 90 90	57.93 57.93 150.44 90 90 90	57.92 57.92 150.45 90 90 90
Total reflections	258403 (8136)	305576 (9776)	364889 (18678)	392043 (28239)	406610 (29742)
Unique reflections	28321 (2168)	33725 (2665)	39098 (3308)	40046 (3557)	40733 (3644)
Multiplicity	9.1 (3.8)	9.1 (3.7)	9.3 (5.6)	9.8 (7.9)	10.0 (8.2)
Completeness (%)	84.85 (65.14)	84.14 (66.24)	86.15 (72.78)	88.20 (78.42)	89.73 (80.34)
Mean I/sigma(I)	21.12 (5.38)	19.67 (4.07)	21.02 (4.49)	21.35 (5.06)	19.46 (4.81)
Wilson B-factor (Å²)	17.9	17.46	17.02	16.78	16.57
R-merge	0.0691 (0.1911)	0.0708 (0.2484)	0.0651 (0.289)	0.0664 (0.3043)	0.0729 (0.3116)
R-meas	0.0730 (0.2208)	0.0747 (0.2876)	0.0686 (0.3166)	0.0699 (0.3253)	0.0766 (0.3324)
R-pim	0.0227 (0.1083)	0.0232 (0.1419)	0.0210 (0.1249)	0.0212 (0.1110)	0.0230 (0.1122)
CC1/2	0.999 (0.959)	0.999 (0.938)	0.999 (0.939)	0.999 (0.952)	0.999 (0.949)

Supplementary Table 2: Data collection statistics for Thaumatin. Numbers in parentheses refer to the highest resolution data shell.

	Insulin				
Energy (keV)	4	4.25	4.5	4.75	5
Wavelength (Å)	3.099	2.917	2.755	2.610	2.479
Resolution range (Å)	39.11 - 1.99 (2.06 - 1.99)	55.32 - 1.87 (1.94 - 1.87)	55.32 - 1.80 (1.86 - 1.80)	55.33 - 1.8 (1.86 - 1.80)	55.33 - 1.80 (1.86 - 1.80)
Space group	$I\ 2_1\ 3$				
Unit cell dimensions (Å,°)	78.22 78.22 78.22 90 90 90	78.23 78.23 78.23 90 90 90	78.24 78.24 78.24 90 90 90	78.25 78.25 78.25 90 90 90	78.26 78.26 78.26 90 90 90
Total reflections	121565 (3748)	140728 (4656)	171255 (8918)	184712 (13884)	192812 (14503)
Unique reflections	10518 (995)	12349 (1214)	14380 (1469)	14389 (1478)	14393 (1474)
Multiplicity	11.6 (3.8)	11.4 (3.8)	11.9 (6.1)	12.8 (9.4)	13.4 (9.8)
Completeness (%)	99.53 (95.31)	97.45 (95.82)	99.85 (98.65)	99.94 (99.46)	99.60 (96.15)
Mean I/sigma(I)	18.69 (1.89)	15.66 (0.94)	15.67 (0.74)	15.26 (0.61)	12.49 (0.30)
Wilson B-factor (Å²)	37.18	38.48	38.38	39.4	41.46
R-merge	0.0671 (0.4596)	0.0729 (0.7336)	0.0713 (1.045)	0.0764 (1.2590)	0.0934 (1.6040)
R-meas	0.0696 (0.5324)	0.0756 (0.8478)	0.0739 (1.1420)	0.0791 (1.3310)	0.0966 (1.6930)
R-pim	0.0182 (0.2630)	0.0197 (0.4157)	0.0191 (0.4459)	0.0201 (0.4283)	0.0241 (0.5337)
CC1/2	1 (0.752)	1 (0.576)	1 (0.439)	0.999 (0.542)	0.999 (0.406)

Supplementary Table 3: Data collection statistics for Insulin. Numbers in parentheses refer to the highest resolution data shell.

	Lysozyme				
Energy (keV)	4	4.25	4.5	4.75	5
Wavelength (Å)	3.099	2.917	2.755	2.610	2.479
Resolution range (Å)	55.69 - 1.99 (2.06 - 1.99)	55.69 - 1.87 (1.94 -1.87)	55.69 - 1.79 (1.85 - 1.79)	55.69 - 1.79 (1.86 - 1.79)	55.70 - 1.79 (1.86 - 1.79)
Space group	$P\ 4_3\ 2_1\ 2$				
Unit cell dimensions (Å,°)	78.76 78.76 37.02 90 90 90	78.75 78.75 37.01 90 90 90	78.76 78.76 37.02 90 90 90	78.76 78.76 37.02 90 90 90	78.78 78.78 37.02 90 90 90
Total reflections	115688 (3780)	136765 (4459)	162711 (7532)	175474 (12076)	181838 (12677)
Unique reflections	14135 (1199)	16882 (1444)	19467 (1684)	19744 (1824)	19927 (1861)
Multiplicity	8.2 (3.2)	8.1 (3.1)	8.4 (4.5)	8.9 (6.6)	9.1 (6.8)
Completeness (%)	92.98 (78.31)	92.57 (78.48)	93.40 (79.46)	95.12 (87.48)	96.02 (89.17)
Mean I/sigma(I)	14.66 (4.17)	16.92 (4.65)	17.02 (4.68)	18.44 (5.93)	22.45 (8.86)
Wilson B-factor (Å²)	23.79	22.71	21.32	21.54	20.83
R-merge	0.1039 (0.3168)	0.0870 (0.2505)	0.0867 (0.3190)	0.0797 (0.3166)	0.0679 (0.2038)
R-meas	0.1098 (0.3788)	0.0918 (0.3013)	0.0913 (0.3600)	0.0837 (0.3431)	0.0713 (0.2198)
R-pim	0.0345 (0.2042)	0.0284 (0.1644)	0.0279 (0.1616)	0.0252 (0.1292)	0.0211 (0.0806)
CC1/2	0.996 (0.884)	0.997 (0.925)	0.998 (0.926)	0.998 (0.954)	0.998 (0.976)

Supplementary Table 4: Data collection statistics for Lysozyme. Numbers in parentheses refer to the highest resolution data shell.

	LMO4_0	LMO4_0.25	LMO4_0.5	LMO4_1	LMO4_2	LMO4_4
Wavelength (Å)	1.281					
Resolution range (Å)	53.49 - 2.10 (2.14 - 2.10)					
Space group	*P* 3 1 2					
Unit cell dimensions (Å,°)	61.77 61.77 92.77 90 90 120					
Total reflections	119348 (6212)	119217 (6186)	119225 (6189)	119312 (6193)	119449 (6200)	119532 (6196)
Unique reflections	12070 (604)	12068 (601)	12068 (601)	12068 (601)	12068 (601)	12067 (601)
Multiplicity	9.9 (10.3)	9.9 (10.3)	9.9 (10.3)	9.9 (10.3)	9.9 (10.3)	9.9 (10.3)
Completeness (%)	99.9 (99.5)	99.9 (99.5)	99.9 (99.5)	99.9 (99.5)	99.9 (99.5)	99.9 (99.5)
Mean I/sigma(I)	16.6 (1.5)	14.0 (0.9)	12.6 (0.7)	11.1 (0.5)	9.5 (0.4)	7.8 (0.3)
Wilson B-factor (Å²)	34.46	34.12	33.81	32.67	31.77	29.8
R-merge	0.099 (0.770)	0.117 (1.273)	0.129 (1.578)	0.148 (2.130)	0.176 (3.265)	0.218 (3.994)
R-meas	0.105 (0.810)	0.123 (1.340)	0.136 (1.660)	0.156 (2.242)	0.186 (3.436)	0.230 (4.202)
R-pim	0.033 (0.248)	0.039 (0.413)	0.043 (0.511)	0.049 (0.692)	0.059 (1.060)	0.072 (1.296)
CC1/2	0.993 (0.853)	0.992 (0.659)	0.992 (0.614)	0.992 (0.431)	0.991 (0.192)	0.990 (0.178)
Anomalous completeness	99.9 (99.7)	99.9 (99.5)	99.9 (99.6)	99.9 (99.6)	99.9 (99.6)	99.9 (99.6)
Anomalous multiplicity	5.2 (5.3)	5.2 (5.3	5.2 (5.3)	5.2 (5.3)	5.2 (5.3)	5.2 (5.3)
Anomalous correlation	0.654 (0.030)	0.553 (0)	0.568 (0.013)	0.484 (0)	0.435 (0)	0.348 (0.030)
Anomalous slope	0.964	0.808	0.742	0.701	0.638	0.583
Anomalous peak heights (σ)						
ZN1	27.55	24.96	23.23	22.05	20.27	19.4
ZN2	24.47	22.48	21.06	20.07	17.8	16.7
ZN3	21.14	19.41	18.57	17.95	17.6	15.36
ZN4	19.66	18.16	18.07	17.21	16.47	15.22

Supplementary Table 5: LMO4 data collection processing statistics and anomalous peak heights shown for different detector background noise levels (0, 0.25, 0.5, 1, 2, 4). Numbers in parentheses refer to the highest resolution data shell.

	Thaumatin_0	Thaumatin_0.25	Thaumatin_0.5	Thaumatin_1	Thaumatin_2	Thaumatin_4
Wavelength (Å)	2.755					
Resolution range (Å)	150.7 - 1.80 (1.83 - 1.80)					
Space group	$P\ 4_1\ 2_1\ 2$					
Unit cell dimensions (Å,°)	58.02 58.02 150.70 90.00 90.00 90.00					
Total reflections	182073 (3578)	182088 (3576)	182180 (3579)	182271 (3579)	182427 (3583)	182729 (3589)
Unique reflections	23745 (1030)	23746 (1030)	23746 (1030)	23747 (1030)	23748 (1030)	23748 (1030)
Multiplicity	7.7 (3.5)	7.7 (3.5)	7.7 (3.5)	7.7 (3.5)	7.7 (3.5)	7.7 (3.5)
Completeness (%)	96.1 (86.3)	96.1 (86.3)	96.1 (86.3)	96.1 (86.3)	96.1 (86.3)	96.1 (86.3)
Mean I/sigma(I)	42.2 (11.9)	41.7 (11.6)	40.0 (10.4)	38.5 (9.5)	35.1 (7.6)	31.6 (6.1)
Wilson B-factor (Å²)	13.45	13.52	13.57	13.63	13.55	13.51
R-merge	0.045 (0.129)	0.046 (0.137)	0.047 (0.143)	0.048 (0.154)	0.051 (0.178)	0.055 (0.212)
R-meas	0.048 (0.152)	0.049 (0.161)	0.050 (0.168)	0.051 (0.182)	0.054 (0.210)	0.058 (0.250)
R-pim	0.016 (0.079)	0.016 (0.083)	0.016 (0.087)	0.017 (0.094)	0.018 (0.109)	0.020 (0.129)
CC1/2	0.999 (0.976)	0.999 (0.974)	0.999 (0.972)	0.999 (0.968)	0.999 (0.959)	0.999 (0.948)
Anomalous completeness	93.4 (66.1)	93.4 (66.1)	93.4 (66.1)	93.4 (66.1)	93.4 (66.1)	93.5 (66.2)
Anomalous multiplicity	4.2 (2.1)	4.2 (2.1)	4.2 (2.1)	4.2 (2.1)	4.2 (2.1)	4.2 (2.1)
Anomalous correlation	0.558 (0.095)	0.551 (0.083)	0.533 (0.053)	0.509 (0.038)	0.488 (0.020)	0.482 (0.020)
Anomalous slope	1.937	1.95	1.85	1.802	1.606	1.477
Anomalous peak heights (σ)						
CYS9	23.56	23.19	22.53	22.02	21.68	20.36
CYS56	22.84	22.66	21.98	21.78	21.43	19.65
MET112	22.17	21.93	21.58	20.93	19.76	19
CYS145	21.49	21.11	20.71	19.68	19.26	18.25
CYS149	20.77	20.5	20.38	19.56	18.74	18.16
CYS77	20.44	20.39	19.96	19.02	18.4	18.08
CYS134	20.43	19.74	19.85	18.95	18.19	17.91
CYS204	19.77	19.41	18.75	18.37	17.44	16.87
CYS193	18.77	18.45	18.52	18.24	17.4	16.8
CYS164	17.47	17.22	17.36	16.78	15.92	15.84
CYS158	17.23	16.8	16.73	16.14	15.89	15.3
CYS66	17.23	16.73	16.5	15.9	15.7	14.99
CYS121	16.66	16.41	16.16	15.89	15.54	14.57
CYS126	16.39	15.99	15.96	15.29	15.3	14.43
CYS71	16.06	15.82	15.15	14.97	14.92	14.01
CYS177	14.35	14.4	14.45	13.96	13.98	13.5
CYS159	10.34	10.31	9.78	9.65	8.96	8.55

Supplementary Table 6: Thaumatin data collection processing statistics and anomalous peak heights shown for different detector background noise levels for thaumatin (0, 0.25, 0.5, 1, 2, 4). Numbers in parentheses refer to the highest resolution data shell.

	RNase A	Petase	ThcOx	SseK3	PAS	Seb1-RRM	AcrB	McjD	$A_{2A}R$	mPGES
Wavelength (Å)	2.755	2.455	3.09	2.755	3.09	2.755	3.024	2.755	2.755	2.755
Resolution range (Å)	41.10-1.80 (1.86-1.80)	67.32-1.70 (1.76-1.70)	74.06-3.20 (3.31-3.20)	49.88-2.10 (2.17-2.10)	72.28-2.50 (2.59-2.50)	54.87-1.77 (1.83-1.77)	28.94-3.40 (3.52-3.40)	54.66-2.80 (2.9-2.80)	89.82-2.40 (2.48-2.40)	45.07-1.77 (1.83-1.77)
Space group	$C\ 2$	$C\ 2\ 2\ 2_1$	$P\ 4_1\ 2_1\ 2$	$P\ 2_1$	$P\ 2_1\ 2_1\ 2$	$C\ 2$	$R\ 3\ 2$	$P\ 2_1\ 2_1\ 2_1$	$C\ 2\ 2\ 2_1$	$R\ 3$
Unit cell (Å,º)	99.98 32.62 72.49 90 90.16 90	52.56 234.31 164.53 90 90 90	111.39 111.39 217.52 90 90 90	96.08 75.02 101.89 90 109.51 90	54.91 144.56 48.65 90 90 90	111.10 46.90 32.30 90 99 90	145.30 145.30 516.84 90 90 120	81.19 109.32 233.35 90 90 90	39.31 179.64 139.54 90 90 90	77.20 77.20 122.06 90 90 120
Total reflections	315464 (11569)	929649 (68871)	552822 (43942)	1430296 (110092)	613436 (48055)	63471 (2083)	2555066 (211481)	2830105 (215179)	941156 (76379)	456856 (16728)
Unique reflections	17977 (1412)	95266 (8111)	21407 (1817)	62053 (4943)	12470 (1065)	10445 (653)	29410 (2892)	51873 (5092)	19968 (1997)	26090 (2383)
Multiplicity	17.5 (8.2)	9.8 (8.4)	25.8 (24.2)	23.0 (22.3)	49.2 (45.1)	6.1 (3.2)	86.9 (73.1)	54.6 (42.3)	47.1 (38.2)	17.5 (7.0)
Completeness (%)	81.29 (65.04)	84.90 (73.12)	91.63 (79.76)	77.31 (60.81)	81.04 (78.66)	64.89 (41.43)	99.70 (99.90)	96.80 (86.27)	99.97 (99.95)	97.51 (75.83)
Mean I/sigma(I)	24.67 (5.03)	18.02 (1.44)	29.98 (2.10)	13.34 (1.79)	41.69 (2.39)	22.91 (3.26)	31.15 (0.97)	12.48 (0.27)	18.63 (0.99)	21.23 (0.48)
Wilson B-factor (Å²)	27.82	30.34	134.61	36.18	64.86	23.2	148.42	105.17	54.09	36.09
R-merge	0.0929 (0.3414)	0.0638 (1.1350)	0.0715 (1.5970)	0.1655 (1.7960)	0.0829 (1.4330)	0.0451 (0.2519)	0.1595 (5.3240)	0.2146 (12.0700)	0.2115 (3.602)	0.0820 (3.4900)
R-meas	0.0954 (0.3644)	0.0672 (1.2110)	0.0729 (1.6310)	0.1693 (1.8400)	0.0838 (1.4500)	0.0491 (0.2998)	0.1604 (5.3610)	0.2167 (12.2100)	0.2138 (3.6480)	0.0840 (3.7900)
R-pim	0.0210 (0.1200)	0.0204 (0.4067)	0.0141 (0.3212)	0.0348 (0.3808)	0.0117 (0.2083)	0.0189 (0.1586)	0.0172 (0.6205)	0.02965 (1.834)	0.03035 (0.5679)	0.0178 (1.4140)
CC1/2	0.995 (0.915)	0.999 (0.837)	0.999 (0.722)	0.997 (0.703)	0.998 (0.636)	0.999 (0.919)	1 (0.52)	0.998 (0.675)	0.999 (0.518)	0.999 (0.21)
CC*	0.999 (0.977)	1 (0.955)	1 (0.916)	0.999 (0.909)	1 (0.882)	1 (0.979)	1 (0.827)	1 (0.898)	1 (0.826)	1 (0.59)
Reflections for refinement	17964 (1410)	95080 (8099)	21403 (1817)	62053 (4831)	11378 (1065)	10395 (650)	29401 (2891)	50341 (4424)	19963 (1997)	25682 (1995)
Reflections for R-free	901 (65)	4672 (409)	2000 (169)	2963 (227)	549 (53)	545 (28)	1501 (154)	2491 (212)	1032 (91)	1255 (112)
R-work	0.2253 (0.3241)	0.1935 (0.4192)	0.2021 (0.3553)	0.2519 (0.4451)	0.2384 (0.3163)	0.1547 (0.1878)	0.3010 (0.4131)	0.2750 (0.3236)	0.2224 (0.3244)	0.1942 (0.4811)
R-free	0.2556 (0.3645)	0.1979 (0.3926)	0.2311 (0.3991)	0.2932 (0.4323)	0.2759 (0.3938)	0.1883 (0.2685)	0.3100 (0.4527)	0.2950 (0.3405)	0.2608 (0.4005)	0.2126 (0.4706)
CC(work)	0.929 (0.816)	0.963 (0.889)	0.951 (0.722)	0.939 (0.712)	0.947 (0.726)	0.965 (0.896)	0.856 (0.475)	0.809 (0.675)	0.797 (0.689)	0.920 (0.373)
CC(free)	0.944 (0.667)	0.957 (0.898)	0.961 (0.652)	0.931 (0.714)	0.939 (0.195)	0.956 (0.716)	0.765 (0.366)	0.610 (0.733)	0.968 (0.508)	0.896 (0.381)
Number of non-H atoms	2035	6622	7289	9913	2674	1277	7854	9060	3321	1250
RMS(bonds/angles) (Å,º)	0.009/1.52	0.004/1.01	0.002/0.49	0.008/1.30	0.009/1.15	0.010/1.09	0.009/1.01	0.009/0.98	0.008/1.21	0.010/1.03
Ramachandran* (%)	97.45/2.55/0	97.83/2.17/0	96.69/3.2/0.11	98.44/1.56/0	100/0/0	96.67/3.33/0	96.22/3.29/0.6	98.13/1.87/0	98.18/1.82/0	99.31/0.69/0
Rotamer outliers (%)	0	0	0.76	1.13	1.71	0	0.6	0.48	0.96	0
Clashscore	3.53	1.23	4.81	4.55	3.99	2.93	8.14	7.27	3.24	5.28
Average B-factor (Å²)	25.06	36.19	140.41	46.67	60.4	24.27	180.06	164.68	61.98	31.95
PDB code	8PX0	6EQD	8PZ5	8PX1	8PX4	8PX5	8PX7	8PX9	8PWN	8PYV

Supplementary Table 7: Data collection and processing statistics for S-SAD experiments. **favored/allowed/outliers.* Numbers in parentheses refer to the highest resolution data shell.

	Fap1 (Ca,S)	AlgE (Ca,S)	NaK2K (K)	Streptactin (Cl)	SERCA (V)	TauA (I)	GP1 (Cd)	RNA G (K)	IRF4 (P,S)
Wavelength (Å)	3.066	2.755	3.350	2.755	2.260	2.375	2.755	3.351	2.755
Resolution range (Å)	65.53-1.97 (2.04-1.97)	56.58-1.77 (1.83-1.77)	48.13-2.26 (2.34-2.26)	54.47-1.80 (1.86-1.80)	64.59-3.13 (3.24-3.13)	65.47-1.77 (1.83-1.77)	46.26-2.75 (2.84-2.75)	28.47-2.19 (2.27-2.192)	64.02-2.60 (2.69-2.60)
Space group	C 2	*C* 2	*I* 4	*I* 4_1 2 2	*P* 2_1	*P* 2_1 2_1 2_1	*P* 6_3 2 2	*P* 2	*C* 2 2 2_1
Unit cell (Å,º)	132.81 30.40 90.60 90 127.04 90	57.35 74.69 115.9 90 102.48 90	68.07 68.07 89.38 90 90 90	57.42 57.42 172.05 90 90 90	131.14 94.39 135.11 90 107.05 90	45.01 71.32 165.03 90 90 90	106.83 106.83 74.86 90 90 120	29.87 23.66 31.06 90 107.61 90	77.88 112.44 140.66 90 90 90
Total reflections	222112 (7826)	1206282 (44200)	152313 (11328)	601286 (22212)	4339345 (388923)	877552 (64950)	207964 (17438)	16129 (632)	1561923 (123304)
Unique reflections	17626 (1321)	45199 (4019)	9459 (915)	13199 (1190)	55956 (5572)	45258 (3837)	6783 (608)	2064 (178)	19282 (1865)
Multiplicity	12.6 (5.9)	26.7 (11.0)	16.1 (12.4)	45.6 (18.7)	77.5 (69.8)	19.4 (16.9)	30.7 (28.7)	7.8 (3.6)	81.0 (66.1)
Completeness (%)	84.33 (64.41)	96.76 (85.58)	98.82 (96.11)	95.25 (87.22)	99.92 (99.86)	85.68 (73.52)	97.20 (90.21)	91.86 (78.76)	99.24 (98.10)
Mean I/sigma(I)	20.29 (8.67)	27.32 (2.51)	42.21 (14.82)	35.10 (3.29)	20.96 (1.04)	15.63 (2.65)	21.54 (0.88)	14.00 (3.25)	41.49 (1.65)
Wilson B-factor (Å²)	14.73	23.68	35.16	21.71	98.58	27.89	102.96	46.43	79.21
R-merge	0.1122 (0.1941)	0.0880 (0.8345)	0.0559 (0.1548)	0.0915 (0.9932)	0.2977 (4.4450)	0.1341 (0.8778)	0.1404 (3.4180)	0.1120 (0.3353)	0.1029 (2.7600)
R-meas	0.1169 (0.2123)	0.0895 (0.8775)	0.0575 (0.1617)	0.0924 (1.0210)	0.2997 (4.4770)	0.1376 (0.9050)	0.1428 (3.4810)	0.1193 (0.3943)	0.1035 (2.7810)
R-pim	0.0309 (0.0808)	0.0156 (0.2598)	0.0132 (0.0455)	0.0127 (0.2268)	0.0339 (0.5261)	0.0297 (0.2118)	0.0252 (0.6358)	0.0392 (0.2016)	0.0109 (0.3168)
CC1/2	0.99 (0.962)	0.999 (0.903)	0.995 (0.99)	1 (0.84)	0.968 (0.642)	0.997 (0.777)	0.999 (0.567)	0.994 (0.857)	1 (0.75)
CC*	0.998 (0.99)	1 (0.974)	0.999 (0.997)	1 (0.955)	0.992 (0.884)	0.999 (0.935)	1 (0.851)	0.998 (0.961)	1 (0.926)
Reflections for refinement	17622 (1321)	45084 (3964)	9457 (915)	13196 (1188)	55923 (5564)	45248 (3837)	6764 (599)	2053 (178)	19219 (1862)
Reflections for R-free	900 (73)	1987 (178)	478 (37)	620 (51)	2815 (287)	2251 (191)	352 (16)	124 (7)	906 (77)
R-work	0.1832 (0.2181)	0.1811 (0.2998)	0.1817 (0.1682)	0.1737 (0.3225)	0.2053 (0.3846)	0.2102 (0.4207)	0.2449 (0.5652)	0.2292 (0.3093)	0.2237 (0.4512)
R-free	0.2229 (0.2600)	0.1906 (0.2732)	0.2233 (0.2240)	0.1936 (0.3715)	0.2607 (0.4354)	0.2319 (0.4379)	0.2829 (0.5122)	0.2750 (0.4517)	0.2499 (0.4797)
CC(work)	0.947 (0.911)	0.962 (0.883)	0.939 (0.956)	0.946 (0.843)	0.905 (0.744)	0.951 (0.819)	0.937 (0.682)	0.959 (0.806)	0.956 (0.749)
CC(free)	0.925 (0.922)	0.958 (0.877)	0.956 (0.910)	0.922 (0.734)	0.864 (0.728)	0.945 (0.824)	0.944 (0.203)	0.929 (0.405)	0.919 (0.542)
Number of non-H atoms	2211	4134	1542	1053	15376	4848	1218	410	3666
RMS(bonds/angles) (Å,º)	0.011/1.58	0.006/0.86	0.015/1.35	0.008/1.21	0.005/1.05	0.010/1.6	0.006/1.03	0.008/1.29	0.01/1.25
Ramachandran* (%)	96.8/3.2/0	96.54/3.46/0	98.91/1.09/0	94.26/5.74/0	94.67/5.02/0.31	98.65/1.01/0.34	97.92/2.08/0	n/a	98.48/1.52/0
Rotamer outliers (%)	0.46	0	1.19	1.02	4.94	0.62	2.99	n/a	0.34
Clashscore	1.29	4.97	5.48	4.68	13.15	3.6	7.59	15.6	4.2
Average B-factor (Å²)	17.5	33.99	39.42	21.35	124.48	28.81	120.12	57.35	115.84
PDB code	8PXC	8PZ4	6DZ1	8PXG	6YSO	8PXH	8PXJ	6JJH	7O56

Supplementary Table 8: Data collection and processing statistics for non S-SAD experiments. The anomalous scatterer used for phasing is indicated in parenthesis in the first row. **favored/allowed/outliers.* Numbers in parentheses refer to the highest resolution data shell.

	OmpK36 (S)	BphA4 (P)	BphA4
Wavelength (Å)	4.133	5.767	1.378
Resolution range (Å)	82.80-2.70 (2.79- 2.70)	76.07-3.77 (3.90-3.77)	38.06-1.60 (1.65-1.60)
Space group	*C* 2	*P* 6_1 2 2	*P* 6_1 2 2
Unit cell (Å,º)	233.03 74.36 90.12 90 112.04 90	98.19 98.19 170.29 90 90 120	98.24 98.24 170.48 90 90 120
Total reflections	860804 (33410)	54693 (1834)	1151500 (98013)
Unique reflections	38517 (3499)	4614 (373)	63940 (6081)
Multiplicity	22.3 (9.5)	11.9 (4.9)	18.0 (16.1)
Completeness (%)	97.40 (89.46)	86.62 (74.45)	98.90 (95.69)
Mean I/sigma(I)	24.03 (4.69)	21.38 (12.17)	23.51 (0.76)
Wilson B-factor (Å^2)	48.84	33.9	29.47
R-merge	0.1001 (0.3662)	0.1448 (0.1385)	0.0656 (2.1020)
R-meas	0.1025 (0.3860)	0.1507 (0.1529)	0.0675 (2.1700)
R-pim	0.0208 (0.1160)	0.0401 (0.0630)	0.0155 (0.5286)
CC1/2	0.996 (0.957)	0.995 (0.987)	1 (0.467)
CC*	0.999 (0.989)	0.999 (0.997)	1 (0.798)
Reflections for refinement	38449 (3498)	4614 (373)	63940 (6066)
Reflections for R-free	1987 (186)	228 (23)	3150 (322)
R-work	0.1987 (0.2649)	0.1853 (0.2194)	0.1777 (0.3583)
R-free	0.2410 (0.2985)	0.1907 (0.2878)	0.2061 (0.3729)
CC(work)	0.920 (0.880)	0.937 (0.898)	0.963 (0.686)
CC(free)	0.914 (0.814)	0.929 (0.861)	0.952 (0.635)
Number of non-H atoms	8278	3058	3422
RMS(bonds/angles) (Å,º)	0.002/0.53	0.01/1.60	0.009/1.54
Ramachandran* (%)	95.34/4.66/0	97.76/2.24/0	98/2/2
Rotamer outliers (%)	0.97	0	0.32
Clashscore	4.5	2.61	2.23
Average B-factor (Å^2)	53.92	38.56	29.1
PDB code	8PYZ	8PXK	8PXL

Supplementary Table 9: Data collection and processing statistics for SAD experiments on laser shaped crystals. **favored/allowed/outliers*. The anomalous scatterer used for phasing is indicated in parenthesis in the first row. Numbers in parentheses refer to the highest resolution data shell.

	RNase A	Petase	ThcOx	Ssek3	PAS	Seb1-RRM	AcrB	McjD	$A_{2A}R$	mPGES	OmpK36
Scatterer	**S**										
1	**15.54**	**23.17**	**16.01**	**16.01**	**19.83**	**20.73**	**15.74**	**13.97**	**11.81**	**19.92**	**34.54**
2	**14.65**	**21.75**	**15.78**	**15.78**	**15.8**	**20.72**	**14.17**	**11.81**	**11.21**	**19.13**	**31.8**
3	**14.51**	**21.67**	**15.74**	**15.74**	**12.11**	**17.07**	**14.08**	**10.82**	**10.84**	**18.64**	**27.29**
4	**14.36**	**21.38**	**15.17**	**15.17**		**16.44**	**12.01**	**10.43**	**10.77**	**18.62**	**21.34**
5	**13.83**	**20.9**	**14.82**	**14.82**		**10.61**	**10.83**	**10.28**	9.65	**18.14**	**11.86**
6	**13.52**	**20.75**	**14.78**	**14.78**			**10.51**	**10.24**	9.64	**15.5**	**10.53**
7	**13.51**	**20.59**	**14.75**	**14.75**			**10.22**	9.6	9.59	**14.64**	**10.07**
8	**13.41**	**19.12**	**14.58**	**14.58**			9.81	9.55	9.42	**13.52**	
9	**13.34**	**18.99**	**14.57**	**14.57**			9.46	9.46	9.4	7.01	
10	**13.33**	**18.91**	**14.2**	**14.2**			9.3	9.2	9.27	5.5	
11	**12.82**	**18.86**	**14.07**	**14.07**			8.42	9.07	8.93		
12	**12.2**	**18.82**	**14.03**	**14.03**			8.25	8.71	8.91		
13	**12.09**	**18.7**	**14.01**	**14.01**			8.1	8.63	8.88		
14	**11.81**	**18.52**	**13.89**	**13.89**			7.88	8.41	8.71		
15	**11.53**	**18.23**	**13.66**	**13.66**			7.82	8.33	8.31		
16	**10.97**	**18.23**	**13.1**	**13.1**			7.12	8.3	7.46		
17	**10.93**	**18.02**	**13.07**	**13.07**			7.1	8.3	6.19		
18	**10.64**	**16.96**	**12.81**	**12.81**			6.99	8.3	5.78		
19	**10.53**	**16.7**	**12.71**	**12.71**			6.4	8.3			
20	9.73	**16.57**	**12.43**	**12.43**			6.02	8.27			
21	8.68	**16.42**	**12.32**	**12.32**			5.91	8.08			
22	7.77	**15.96**	**12.18**	**12.18**			5.73	7.95			
23	7.14	**15.92**	**12.1**	**12.1**			5.56	7.94			
24	6.67	**15.87**	**11.62**	**11.62**			5.47	7.8			
25		**15.35**	**11.62**	**11.62**			5.36	7.77			
26		**15.3**	**11.09**	**11.09**			5.35	7.77			
27		**15.27**	**10.98**	**10.98**			5.33	7.51			
28		**14.87**	**10.8**	**10.8**			5.2	7.12			
29		**14.44**	**10.78**	**10.78**			5.15	6.77			
30		**13.3**	**10.58**	**10.58**			5.11	6.6			
31			**10.53**	**10.53**			5.03	6.49			
32			**10.24**	**10.24**			5.03	5.74			
33			9.93	9.93			5.03	5.24			
34			9.22	9.22			4.98	5.19			
35			8.95	8.95			4.95	5.16			
36			8.57	8.57			4.92	5			
37			8.54	8.54			4.85	4.99			
38			8.39	8.39			4.81	4.95			
39			7.78	7.78			4.79	4.92			
40			7.4	7.4			4.78	4.79			
41							4.77	4.73			
42							4.72	4.66			
43							4.65	4.6			
44							4.64	4.56			
45							4.62				

Supplementary Table 10: Sulfur peak heights (σ) detected by ANODE from phased anomalous difference Fourier maps. Peaks above 10 σ are shown in bold.

	Fap1		AlgE		NaK2K	Streptactin	SERCA				TauA	GP1	RNA G	IRF4		BphA4
Scatterer	Ca	S	Ca	S	K	Cl	V	K	Cl	S*	I	Cd	K	P	S	P
1	**64.16**			**34.5**	**40.95**	**21.69**	**42.98**				**75.39**	**22.82**	**13.89**		**18.4**	**39.71**
2	**45.45**			**31.8**	**39.19**	**18.22**	**37.58**				**52.58**	**19.76**	**10.62**	**13.49**		
3		**11.08**		**27.3**	**38.6**	**17.03**		**11.65**			**34.87**	**18.21**	**10.45**		**13.03**	
4				**21.3**	**37.95**	**11.53**			8.74		**32.51**	**15.97**		**12.74**		
5			**11.86**		**36.42**	8.79		8.69			**29.38**	**12.93**		**12.68**		
6			**10.53**		**36.27**	8.75				8.15	**16.44**			**12.63**		
7				**10.1**	**31.23**	8.02				7.81	**14.05**			**12.23**		
8					**27.83**					7.56	**13.05**			**12.01**		
9					**10.33**					7.44				**11.06**		
10					6.75					7.38				9.97		
11										7.36				9.45		
12										7.18				9.4		
13										7.14				8.16		
14										7.09				7.98		
15										6.77				7.92		
16										6.69				7.87		
17										6.68				7.46		
18										6.53				7.34		
19										6.46				7.32		
20										6.44				7.32		
21										6.39				7.28		
22										6.37				6.77		
23										6.37				6.7		
24										6.34				5.99		
25									6.32					5.96		
26										6.26				5.91		
27										6.26				5.58		
28										6.22				5.39		
29										6.21				5.39		
30										6.07				5.37		
31										6.05				5.33		
32										6.02				5		
33										6.01				4.96		
34										6				4.85		
35										5.99					4.73	
36										5.95				4.5		
37										5.95				4.39		
38										5.83				4.38		
39										5.79				4.38		

Supplementary Table 11: Peak heights (σ) detected by ANODE from phased anomalous difference Fourier maps. Peaks above 10 σ are shown in bold. * Only 39 of 80 peaks are shown for S.

```python
import binascii
import sys
import os
from multiprocessing import Pool, cpu_count
from cbflib_adaptbx import uncompress, compress
from scitbx.random import variate, poisson_distribution

start_tag = binascii.unhexlify("0c1a04d5")
rate = float(os.environ.get("RATE", "0.0"))

if rate <= 0:
    sys.exit(f"Impossible rate constant: {rate}")

def add_noise(filename_in):
    filename_out = f"noisy_{filename_in}"

    print(f"{filename_in} -> {filename_out}")

    data = open(filename_in, "rb").read()

    generator = variate(poisson_distribution(mean=rate))
    data_offset = data.find(start_tag) + 4
    cbf_header = data[: data_offset - 4]

    fast = 0
    slow = 0
    length = 0

    for record in cbf_header.decode().split("\n"):
        if "X-Binary-Size-Fastest-Dimension" in record:
            fast = int(record.split()[-1])
        elif "X-Binary-Size-Second-Dimension" in record:
            slow = int(record.split()[-1])
        elif "X-Binary-Number-of-Elements" in record:
            length = int(record.split()[-1])
        elif "X-Binary-Size:" in record:
            size = int(record.split()[-1])

    assert length == fast * slow

    pixel_values = uncompress(
        packed=data[data_offset : data_offset + size], fast=fast, slow=slow
    )

    tailer = data[data_offset + 4 + size :]

    for j in range(len(pixel_values)):
        if pixel_values[j] < 0:
            continue
        pixel_values[j] += next(generator)

    compressed = compress(pixel_values)

    fixed_header = ""

    for record in cbf_header.decode().split("\n"):
        if "Content-MD5" in record:
            pass
        elif "X-Binary-Size:" in record:
            fixed_header += f"X-Binary-Size: {len(compressed)}\r\n"
        else:
            fixed_header += f"{record}\n"

    with open(filename_out, "wb") as f:
        f.write(fixed_header.encode())
        f.write(start_tag)
        f.write(compressed)
        f.write(tailer)

if __name__ == "__main__":
    p = Pool(cpu_count())
    p.map(add_noise, sys.argv[1:])
```

Supplementary Note 1: Python script for adding detector background noise. The script adds a random value drawn from a Poisson distribution to every detector pixel.
